# Persistent sheaf Laplacian analysis of protein stability and solubility changes upon mutation

**DOI:** 10.1002/pro.70700

**Published:** 2026-07-10

**Authors:** Yiming Ren, Junjie Wee, Xi Chen, Grace Qian, Guo‐Wei Wei

**Affiliations:** ^1^ Department of Mathematics Michigan State University East Lansing Michigan USA; ^2^ The Frazer School Gainesville Florida USA; ^3^ Lassiter High School Marietta Georgia USA; ^4^ Department of Mathematics University of Georgia Athens Georgia USA; ^5^ Department of Biochemistry and Molecular Biology University of Georgia Athens Georgia USA

**Keywords:** mutation, persistent topological Laplacians, protein folding stability, protein solubility, sheaf Laplacian networks

## Abstract

Genetic mutations frequently disrupt protein structure, stability, and solubility, acting as primary drivers for a wide spectrum of diseases. Despite the critical importance of these molecular alterations, existing computational models often lack interpretability and fail to integrate essential physicochemical interactions. To overcome these limitations, we propose SheafLapNet, a predictive framework grounded in the mathematical theory of Topological Deep Learning (TDL) and Persistent Sheaf Laplacian (PSL). Unlike standard Topological Data Analysis (TDA) tools such as persistent homology, which are often insensitive to heterogeneous information, PSL explicitly encodes specific physical and chemical information such as partial charges directly into the topological analysis. SheafLapNet synergizes these sheaf‐theoretic invariants with advanced protein transformer features and auxiliary physical descriptors to capture intrinsic molecular interactions in a multiscale and mechanistic manner. To validate our framework, we employ rigorous benchmarks for both regression and classification tasks. For stability prediction, we utilize the comprehensive S2648 dataset, alongside the independent S350 and strictly non‐redundant S669 blind test sets to ensure robust evaluation and thermodynamic consistency. For solubility prediction, we employ the PON‐Sol2 dataset, which provides annotations for increased, decreased, or neutral solubility changes. By integrating these multi‐perspective features, SheafLapNet achieves state‐of‐the‐art performance across these diverse benchmarks, demonstrating that sheaf‐theoretic modeling significantly enhances both interpretability and generalizability in predicting mutation‐induced structural and functional changes.

## 1 INTRODUCTION

Genetic mutations induce alterations in the amino acid sequence that can severely disrupt the delicate thermodynamic balance of protein structures. These atomic‐level perturbations frequently compromise critical physicochemical attributes, including folding stability, binding affinity, reactivity, and solubility, often precipitating loss‐of‐function phenotypes or toxic aggregation linked to severe pathologies. Such molecular mechanisms are central to the etiology of neurodegenerative disorders like Alzheimer's (Hurley et al., 2023) and Parkinson's disease (Funayama et al., 2023), as well as various cancers (Chen, Zhang, et al., 2022) and metabolic syndromes (Zhang et al., 2011; Zhang et al., 2013). Despite the clinical criticality of these stability and solubility profiles, accurately predicting mutation‐induced changes remains a formidable challenge. Protein solubility is governed by a multifaceted interplay of factors, ranging from intrinsic sequence motifs and post‐translational modifications to extrinsic environmental conditions such as solvent type, ionic strength, and temperature. This complexity is compounded by the sheer volume of genomic data; while more than four million missense variants have been cataloged, the majority remain classified as variants of uncertain significance (VUS) (Karczewski et al., 2020), and existing datasets often lack the comprehensive environmental annotations required to resolve these nuances. Given that traditional experimental validation remains resource‐intensive and low‐throughput (Marian, 2020; Molotkov et al., 2024), there is an urgent need for advanced computational approaches capable of decoding these complex biophysical interactions to reliably predict the functional impact of genetic variants.

Protein stability and solubility are fundamental to protein function and directly involved in human disease, yet predicting mutation‐induced changes in these properties remains a complex computational challenge. In the domain of stability, deep learning architectures have capitalized on large‐scale datasets. For instance, mutDDG‐SSM (Li et al., 2024) hybridizes graph attention networks with gradient boosting trees, while TopologyNet (Cang & Wei, 2017) employs a multi‐task framework to link stability data with disease associations. Models developed over the past decade, such as STRUM (Quan et al., 2016), have leveraged modern machine learning to uncover hidden relationships between structure and stability but often provide limited interpretability. To address modern benchmarking standards, the field has recently witnessed a rapid expansion of advanced methodologies. These contemporary innovations include transfer learning architectures (Dieckhaus et al., 2024), sophisticated sequence‐based embedding models (Savojardo et al., 2025; Yao et al., 2025), novel structure‐aware predictive networks (Sun et al., 2025), and foundational thermodynamic frameworks (Lee et al., 2026). Parallel efforts in the prediction of mutation‐induced solubility changes have yielded tools such as CamSol (Sormanni et al., 2015), SODA (Paladin et al., 2017), Solubis (Van Durme et al., 2016), PON‐Sol (Yang et al., 2016), and PON‐Sol2 (Yang et al., 2021), which utilize gradient boosting on expanded datasets. However, a unified framework capable of simultaneously addressing these coupled properties remains absent. Furthermore, despite their predictive power, many existing models may fail to explicitly account for fundamental physical interactions, including hydrogen bonding, van der Waals forces, hydrophobicity, and electrostatics, that govern molecular behavior (Stefl et al., 2013; Sun et al., 2022). This limitation, coupled with suboptimal performance metrics like the normalized Correct Prediction Ratio in solubility tasks, underscores an urgent demand for Explainable AI frameworks capable of providing interpretable, mechanistically grounded insights into disease causality.

To address these challenges, we turn to Topological Data Analysis (TDA), an emerging mathematical field that utilizes algebraic topology to analyze structural patterns within complex data. Its central tool, persistent homology (Carlsson, 2009; Epstein et al., 2011; Zomorodian & Carlsson, 2004), integrates classical homology and filtration to create a multiscale analysis of data. TDA becomes a powerful approach in data science when it is paired with machine learning. In particular, Topological Deep Learning (TDL) that integrates TDA and deep neural networks was introduced for the first time in 2017 (Cang & Wei, 2017). Over the years, TDL has become a new frontier in rational learning (Papamarkou et al., 2024). Various deep neural networks have been proposed (Barbero et al., 2022; Hajij et al., 2025). TDL has achieved significant success in deciphering biomolecules (Cang et al., 2018). Some of the most compelling application examples in which TDL has consistently demonstrated clear advantages over competing methods include its strong performance in the D3R Grand Challenges (Nguyen et al., 2019), its role in uncovering the evolutionary mechanisms of SARS‐CoV‐2 (Chen, Wang, et al., 2020), and its successful forecasting of viral variants (Chen & Wei, 2022).

However, traditional TDA with persistent homology faces inherent limitations as it is insensitive to homotopic shape evolution without topological changes, cannot distinguish between geometric isomers, and is incapable of differentiating atom types and encoding directed relations. To overcome these limitations, the persistent spectral theory (Wang et al., 2020), also known as the Persistent Laplacian (PL) (Mémoli et al., 2022; Wang et al., 2021), was proposed. By capturing homotopic shape evolution via nonharmonic spectra, computational algorithms such as the HERMES software (Wang et al., 2021), homotopy continuation (Wei & Wei, 2021) and PETLS (Jones & Wei, 2025) have facilitated a new paradigm of Topological deep learning. This approach has demonstrated superior efficacy across a range of biomolecular tasks, including protein‐ligand binding affinity prediction (Meng & Xia, 2021), protein–protein interaction analysis (Liu, Feng, et al., 2022; Wee & Xia, 2022), and the early prediction of dominant SARS‐CoV‐2 variants, such as Omicron BA.4 and BA.5, 2 months prior to their announcement by the World Health Organization (WHO) (Chen, Qiu, et al., 2022).

Despite their established utility, neither standard persistent homology nor the PL is able to capture heterogeneous information within complex datasets. To address this limitation, element‐specific persistent homology was originally developed to explicitly distinguish between different atomic types (Cang et al., 2018), an approach that subsequently inspired a wave of novel TDA methodologies (Ameneyro et al., 2024; Grbić et al., 2022; Liu, Feng, et al., 2022; Liu, Xia, et al., 2022). Building on this foundation, Persistent Sheaf Laplacian (PSL) (Wei & Wei, 2025a) has recently emerged as a more mathematically elegant theory capable of embedding heterogeneous information, such as geometry and partial charges, directly into topological analysis via the theory of cellular sheaves (Curry, 2014; Hansen & Ghrist, 2019). Distinct from standard Laplacians, this sheaf‐theoretic framework allows the assignment of specific vector spaces, representing chemical properties, to the open sets of protein topology (Wei & Wei, 2025a). By associating specific weights with atoms (nodes), PSL encodes local topological and geometric information directly into its harmonic and non‐harmonic spectra, thereby enabling the precise capture of intrinsic mutation‐induced physical and chemical interactions. A review of persistent topological Laplacians is available in (Wei & Wei, 2025b). TDA approaches beyond persistent homology are surveyed in (Su et al., 2026). Recent advances in TDL are reviewed in (Papamarkou et al., 2024). For application perspectives, the reader is further referred to a comprehensive review of TDA and TDL in molecular sciences (Wee & Xia, 2022).

In this work, we introduce SheafLapNet, a novel deep learning framework grounded in PSL theory, designed to predict mutation‐induced changes in both protein stability and solubility. The SheafLapNet architecture synergizes three distinct categories of features to construct a comprehensive molecular representation: (1) multiscale topological and geometric features derived from Persistent Sheaf Laplacians, (2) auxiliary physicochemical descriptors capturing local atomic interactions, and (3) evolutionary sequence embeddings extracted from the pre‐trained ESM‐2 protein transformer (Lin et al., 2023). These diverse feature sets are integrated into a unified representational framework, which is subsequently processed by task‐specific neural network architectures to independently predict both stability and solubility alterations. We rigorously evaluated SheafLapNet against established benchmarks. For stability prediction, we employed the S2648 dataset, alongside the independent S350 and strictly non‐redundant S669 blind test sets to evaluate generalization and thermodynamic consistency. For solubility classification, we utilized the PON‐Sol2 dataset. Across these comprehensive assessments, SheafLapNet consistently outperforms existing state‐of‐the‐art models. These results demonstrate a predictive accuracy improvement, validating the efficacy of incorporating sheaf‐theoretical invariants for mutation impact prediction.

## 2 RESULTS

### 2.1 Overview of SheafLapNet

**FIGURE 1:**
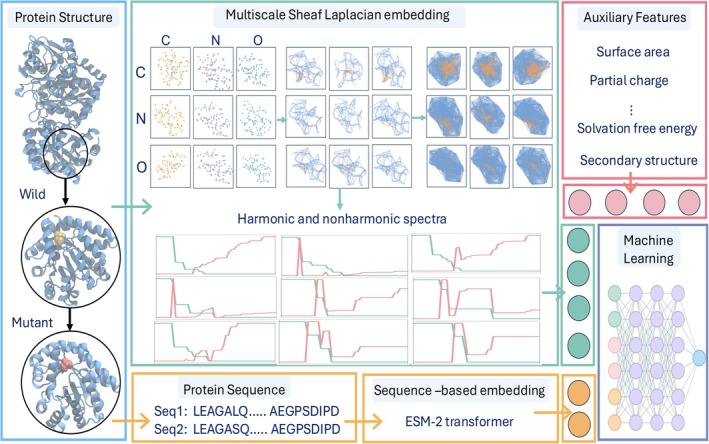
Illustration of the Persistent Sheaf Laplacian (PSL) neural network (SheafLapNet) workflow. The framework predicts mutation‐induced stability and solubility changes by integrating multi‐scale protein representations. For each input protein structure, the feature generation pipeline extracts three distinct components: (1) sequence‐based embeddings derived from pretrained protein Transformer models, (2) topological features computed via the PSL framework, and (3) auxiliary physicochemical features. These three sets of features are concatenated to form the input of the neural network for the prediction task.

Figure 1 outlines the workflow of SheafLapNet. As a standard machine learning model, SheafLapNet extracts features from protein structure and uses a neural network to predict mutation‐induced stability and solubility changes upon mutation. The workflow begins with 3D protein structures from datasets, with corresponding mutant structures generated using the Jackal software (Xiang & Honig, 2002). The feature generation process consists of three components: sequence features from pretrained protein Transformer, topological features from persistent Sheaf Laplacians, and auxiliary physicochemical features. For topological features, atom subsets around the mutational site are extracted from both wild‐type and mutant proteins to form element‐specific subcomplexes. These subcomplexes are utilized to compute the harmonic and nonharmonic spectra of sheaf Laplacians under a structural filtration, creating a Sheaf Laplacian embedding that characterizes atom–atom interactions across multiple scales. For sequence features, the FASTA sequences of the wild‐type and mutant proteins are extracted from the complex and input into the pretrained Transformer models. The derived latent space embeddings are used as the sequence features. For physicochemical features, we consider atom‐level properties such as partial charge, electrostatic solvation free energy, and Coulomb interactions, as well as residue‐level properties such as mutation site neighbor amino acid composition, pKa shifts, and additional physicochemical properties. These three types of feature embeddings are concatenated to form the input feature vector for the machine learning algorithm (neural network) to predict mutation‐induced stability changes and solubility changes. Full details of the neural network architecture, including hidden layer configurations, loss functions, and dataset‐specific hyperparameters, are provided in Table S1. Furthermore, the framework operates with high computational efficiency across all benchmarked datasets, as detailed in Table S8.

### 2.2 Prediction of mutation‐induced protein stability changes

Mutation‐induced perturbations in protein stability are a fundamental mechanism underlying numerous genetic diseases. Accurately predicting these stability alterations, quantified as ΔΔG, requires a rigorous accounting of the complex structural and physicochemical environments surrounding the mutation site. To systematically validate our framework's capacity to capture these intricate relationships, we establish a comprehensive, two‐tiered evaluation protocol. To quantify predictive performance across all downstream tasks, we employ the Pearson correlation coefficient (PCC) and the root mean squared error (RMSE), as defined in Section S1.1. We first emphasize the S669 independent blind test because it provides the most stringent assessment of homology‐free generalization. Subsequently, to establish baseline cross‐validation performance, we comprehensively analyze the model on the standard S2648 training dataset and its associated S350 subset.

#### 2.2.1 Independent blind test evaluation on the S669 dataset

Traditional benchmarks for protein stability prediction often suffer from structural redundancy, where test samples share high sequence identity with the training sets. To ensure a genuinely unbiased evaluation of SheafLapNet's generalization capabilities against other state‐of‐the‐art approaches under strict homology‐free conditions, we utilized the independent S669 benchmark dataset (Pancotti et al., 2022). The S669 dataset comprises 1338 direct and reverse single‐site variations occurring across 95 protein chains, with experimental ΔΔG values retrieved from ThermoMutDB (Xavier et al., 2021) and manually curated. Consistent with established conventions, negative ΔΔG values indicate destabilizing variations. Importantly, the S669 dataset was explicitly constructed to be non‐redundant at a strict 25% sequence identity threshold with respect to widely used training datasets, including VariBench (Nair & Vihinen, 2013) and S2648. For this blind test, we trained our framework on the complete S2648 dataset, which is a standard comprehensive dataset comprising 2648 single‐point mutations across 131 protein structures, maintaining strict alignment with standard benchmarking protocols.

By construction, the S2648 dataset is inherently unbalanced toward destabilizing variations. To mitigate this energetic bias and enhance the model's capacity to accurately predict stabilizing variations, we exploited the physical principle of thermodynamic reversibility, defined as $\Delta \Delta G\left(A\to B\right)=-\Delta \Delta G\left(B\to A\right)$ (Capriotti, Fariselli, Calabrese, & Casadio, 2005). Utilizing this property, the training set was artificially expanded to include reverse variations by inverting the feature representations and the corresponding experimental ΔΔG values.

**FIGURE 2:**
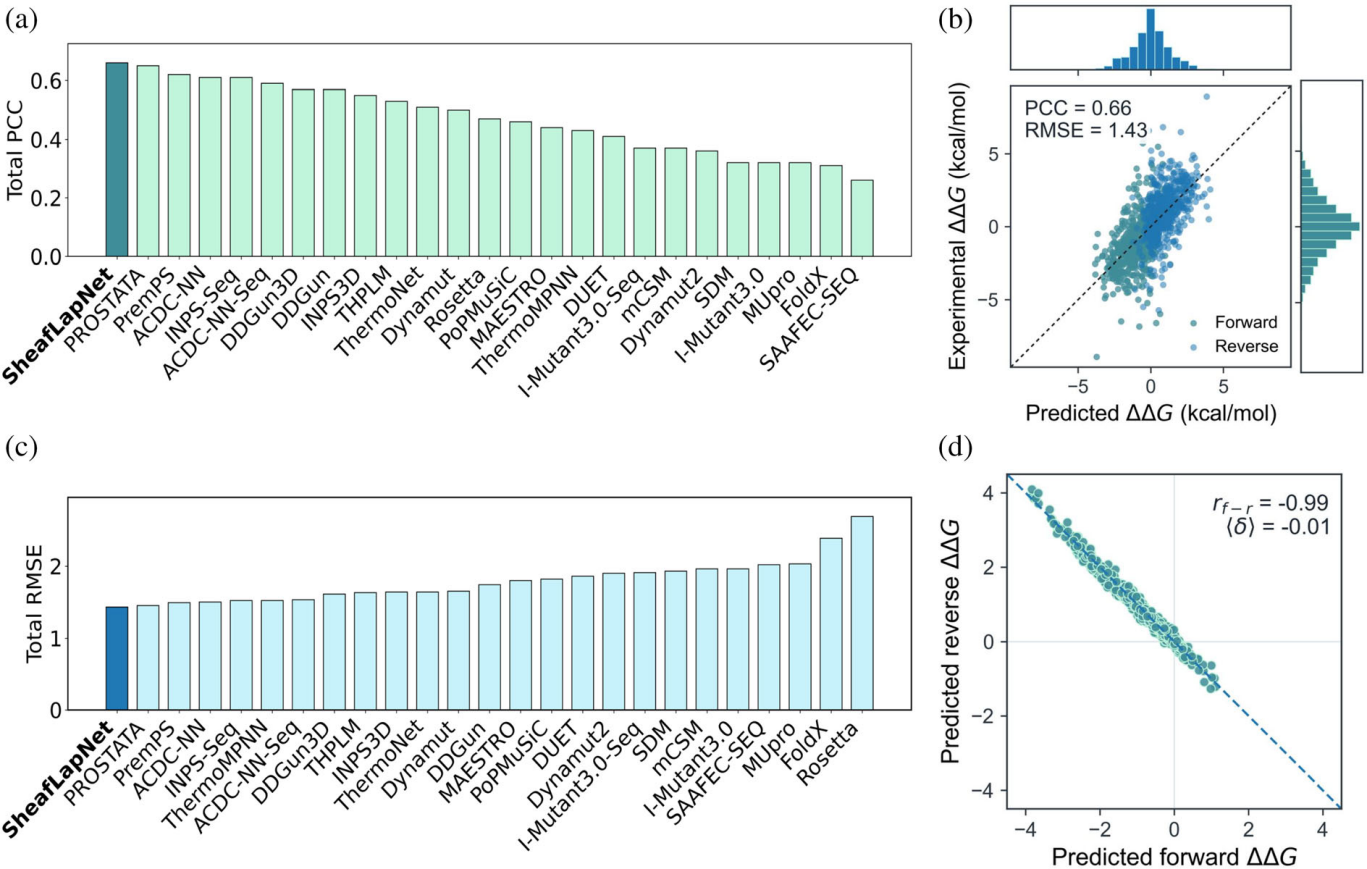
Independent blind test performance and thermodynamic consistency on the S669 dataset. (a) Comparison of the Total PCC between SheafLapNet and various state‐of‐the‐art predictive methods (Benevenuta et al., 2021; Capriotti, Fariselli, & Casadio, 2005; Chen, Lu, et al., 2020; Cheng et al., 2006; Dehouck et al., 2011; Dieckhaus et al., 2024; Fariselli et al., 2015; Gong et al., 2023; Kellogg et al., 2011; Laimer et al., 2015; Laimer et al., 2016; Li et al., 2020; Li et al., 2021; Montanucci et al., 2019; Pancotti et al., 2022; Pires et al., 2014a; Pires et al., 2014b; Rodrigues et al., 2018; Rodrigues et al., 2021; Savojardo et al., 2016; Schymkowitz et al., 2005; Umerenkov et al., 2023; Worth et al., 2011). (b) Scatter plot comparing the experimental versus predicted ΔΔG values for both forward (direct) and reverse mutations evaluated by SheafLapNet. (c) Comparison of the Total RMSE across the evaluated methods. (d) Anti‐symmetry analysis plotting the predicted reverse ΔΔG against the predicted forward ΔΔG. The results demonstrate near‐perfect thermodynamic consistency with an anti‐symmetry correlation of $r_{f-r}=-0.99$ and a bias of $\left\langle \delta \right\rangle =-0.01$.

We benchmarked SheafLapNet against numerous state‐of‐the‐art methods utilizing the S669 dataset, as visually summarized in Figure 2 and detailed in Table S5. For a comprehensive performance assessment, we report the PCC and RMSE evaluated under three distinct settings: (i) considering all variations, both forward and reverse (Total), (ii) strictly isolating direct variations (Forward), and (iii) strictly isolating reverse variations (Reverse). Furthermore, to evaluate the thermodynamic consistency of the predictive models, we computed the anti‐symmetry correlation ($r_{f-r}$) and the anti‐symmetry bias ($\left\langle \delta \right\rangle$) as defined in Section S1.1. As demonstrated in Figure 2a,c, SheafLapNet achieves competitive performance across the evaluated metrics, establishing it among the leading models for mutation analysis. When considering the Total dataset combining forward and reverse mutations, SheafLapNet achieves a PCC of 0.66 and an RMSE of 1.43 kcal/mol, which is further illustrated by the strong concordance between predicted and experimental values shown in Figure 2b. However, The thermodynamic consistency of SheafLapNet is further supported by the anti‐symmetry analysis. As depicted in the scatter plots (Figure 2d), our framework achieves an almost perfect anti‐symmetry correlation of $r_{f-r}=-0.99$ and an anti‐symmetry bias of $\left\langle \delta \right\rangle =-0.01$. Crucially, SheafLapNet maintains highly symmetrical predictive consistency across both directional evaluation splits, attaining a PCC of 0.50 on Forward mutations and 0.48 on reverse mutations. This negligible degradation between direct and inverse predictions confirms that the model is not merely overfitting to the destabilizing bias inherently presented in natural wild‐type structures. Instead, the near‐perfect anti‐symmetry metrics ($\left\langle \delta \right\rangle \approx 0$ and $r_{f-r}\approx -1$) strongly suggest that the integration of topological features, physicochemical descriptors, and transformer‐based sequence embeddings helps enforce thermodynamically consistent predictions. To ensure an impartial evaluation environment, we did not include some recent studies (Barducci et al., 2026; Savojardo et al., 2025) of dataset S669 in our comparison because of their alternative training/validation/test protocols.

#### 2.2.2 Cross‐validation and blind test performance on S2648 and S350

To complement the rigorous S669 blind test and establish our model's foundational cross‐validation baseline, we further analyzed the framework using the standard S2648 benchmark dataset. Our evaluation protocol here consisted of two distinct phases. First, we performed a five‐fold cross‐validation on the complete S2648 dataset to establish baseline generalizability. This was followed by a targeted blind test using the S350 dataset, a high‐quality curated subset of 350 mutations across 67 proteins that has served as a standard benchmark for independent model evaluation.

The predictive efficacy of the Sheaf Laplacian framework is substantiated by the superior performance of SheafLapNet across both validation protocols. In the comprehensive S2648 cross‐validation analysis, we benchmarked our model against existing state‐of‐the‐art methods as shown in Figure S2a. SheafLapNet significantly outperforms established baselines, including STRUM (Quan et al., 2016), and the leading topological convolutional neural network, TNet‐MP‐2 (Cang & Wei, 2017). An extended quantitative benchmark evaluating SheafLapNet against established predictive models on the S2648 and S350 datasets is provided in Table S6. While these methods achieve a PCC of approximately 0.77 and an RMSE of 0.94 kcal/mol, our model demonstrates superior predictive capability, attaining a PCC of 0.82 and an RMSE of 0.84 kcal/mol. This corresponds to a 6.49% improvement in Pearson correlation and a 9.6% reduction in prediction error relative to TNet‐MP‐2. As illustrated in Figure S2c, the scatter plot demonstrates a tight concordance between predicted and experimentally measured stability changes. To transparently address inherent dataset homology, rigorous BLASTp analysis revealed that 99.58% of validation mutations across the S2648 5‐fold splits share >25% sequence identity with their respective training folds. This occurs because the test samples frequently originate from the same proteins present in the training folds.

As a secondary benchmark for comparability with prior studies (Cang & Wei, 2017; Quan et al., 2016; Worth et al., 2011), we conducted a blind test on the S350 benchmark. To establish a rigorous testing protocol, the training dataset for this evaluation was constructed by strictly removing the 350 mutation samples comprising the S350 benchmark from the comprehensive S2648 dataset, resulting in a dedicated training set of 2298 mutations. Because the S350 benchmark frequently evaluates different mutation sites on the exact same proteins present in the training set, our BLASTp alignment confirmed that 346 out of 350 mutations in the S350 test set are located in proteins sharing >25% sequence identity with this dedicated training subset. In this assessment, SheafLapNet maintained high predictive accuracy, achieving an average PCC of 0.82 and an RMSE of 0.90 kcal/mol. As depicted in the comparative analysis in Figure S2b, our model outperforms TNet‐MP‐2, which reports a PCC of 0.81 and an RMSE of 0.94 kcal/mol. A direct comparison between the experimental and predicted ΔΔG values for the test set is presented in Figure S2d. The necessity of synergizing topological invariants, sequence embeddings, and physicochemical descriptors to achieve this high predictive accuracy is rigorously validated through feature ablation studies detailed in Table S2. Beyond predictive accuracy, the PSL framework demonstrates superior robustness regarding data coverage. For instance, I‐Mutant 3.0 failed to evaluate the complete dataset, covering only 2636 of 2648 samples in the S2648 benchmark and 338 of 350 in S350. In contrast, SheafLapNet successfully generated predictions for every mutation sample in both benchmarks. This complete coverage underscores the scalability of Sheaf Laplacian embeddings and highlights their computational reliability.

**FIGURE 3:**
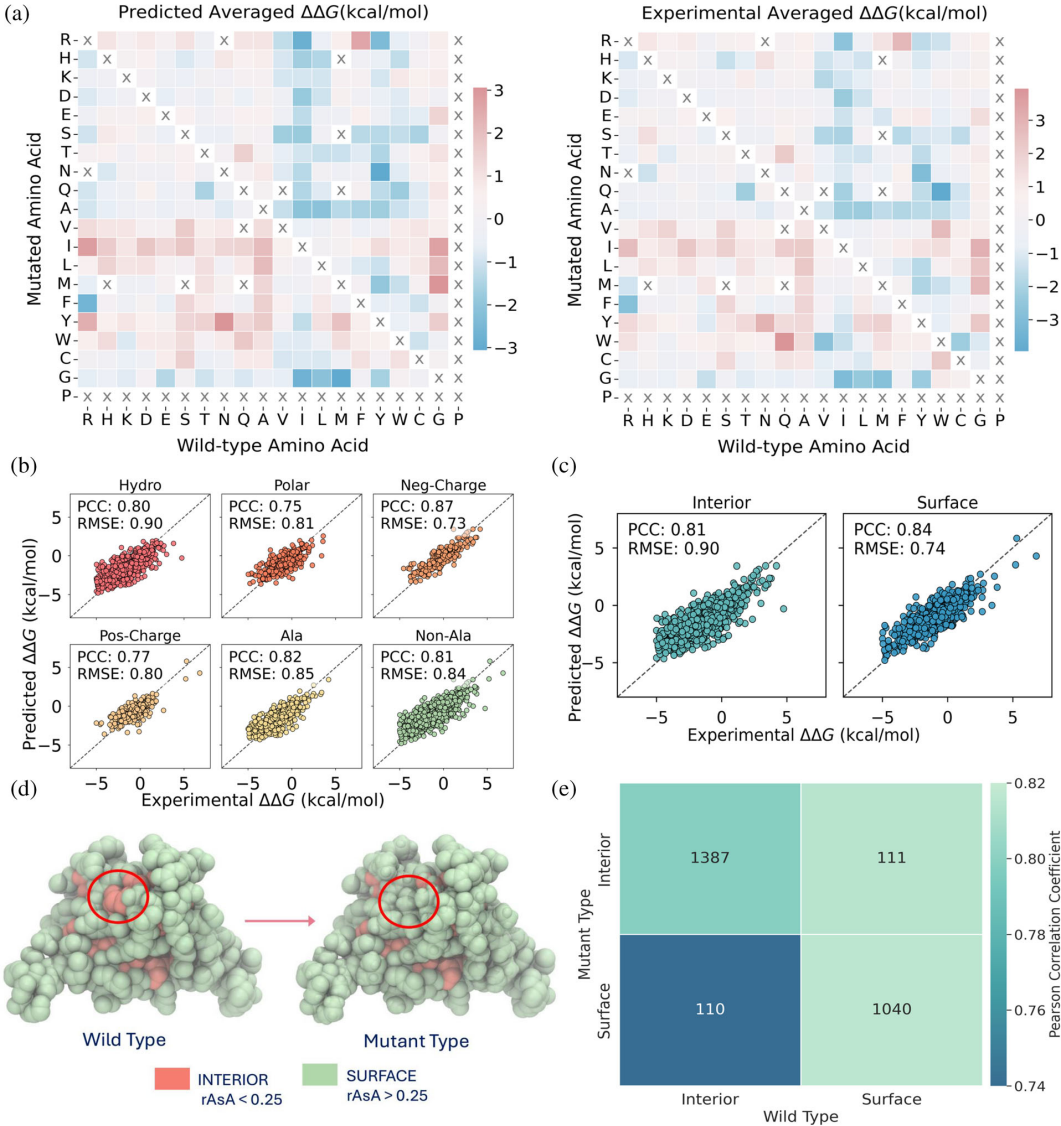
Illustration of the out‐of‐fold model performance derived from a 5‐fold cross‐validation predicting protein stability changes upon mutation in the S2648 dataset. (a) Residue‐residue matrix comparing the averaged experimental and predicted mutation‐induced stability changes ΔΔG for the whole dataset of 2648 mutations, where X indicates no mutation samples. (b) Model performance across different mutation types based on residue physicochemical properties. (c) Model performance across different structural regions defined by the relative accessible solvent area (rASA). (d) Structural shift on protein (PDB ID: 1ARR) of the Q39G mutation from interior to surface region. (e) Model performance stratified by four mutation region types, with cell annotations indicating the sample count per category.

Utilizing strictly out‐of‐fold predictions aggregated from the 5‐fold cross‐validation on the S2648 dataset, we evaluated the concordance between average experimental and predicted stability changes across diverse mutation types. As shown in the Figure 3a, a residue‐residue matrix is established where the x‐axis corresponds to the wild‐type residues and the y‐axis denotes the mutated residues. Each cell at the intersection of a specific mutant row and wild‐type column represents the averaged ΔΔG value for that specific substitution pair, and the corresponding number of mutation samples and MSE matrix is provided in Figure S3 for each substitution. To capture the complete stability landscape, we explicitly incorporated reverse mutations by assigning opposite stability change values, resulting in an antisymmetric matrix structure. The predicted matrix closely mirrors the patterns observed in the experimental benchmark, indicating that the model successfully captures mutation‐specific stability trends. While the overall landscape of perturbations is preserved, the variance in the predicted values appears attenuated compared to the experimental data. A prominent feature emerging from this analysis is that mutations to Isoleucine consistently yield positive ΔΔG values across the majority of wild‐type residues. A possible explanation is that Isoleucine is often unable to compensate for the loss of favorable hydrophilic interactions, such as hydrogen bonds and electrostatic contacts, that are originally contributed by polar or charged wild‐type residues. This leads to a lack of chemical compatibility with the surrounding aqueous solvent and consequently results in positive ΔΔG values.

To further evaluate the sensitivity of the model to specific physicochemical properties, mutation residues were stratified into six distinct categories: hydrophobic, polar, negatively charged, positively charged, alanine, and non‐alanine. The correspondence between predicted and experimental stability changes for each category is presented in Figure 3b. Quantitative analysis reveals consistent predictive performance across all groups. Specifically, the PCC (RMSE) values are 0.80 (0.90 kcal/mol), 0.75 (0.81 kcal/mol), 0.87 (0.73 kcal/mol), 0.77 (0.80 kcal/mol), 0.82 (0.85 kcal/mol), and 0.81 (0.84 kcal/mol) for hydrophobic, polar, negatively charged, positively charged, alanine, and non‐alanine mutations, respectively. Notably, robust results were obtained across all mutation types, indicating that the model generalizes effectively across diverse amino acid substitutions without exhibiting significant bias. The consistently high correlation observed across these varying chemical groups suggests that the underlying feature representation effectively captures the distinct stabilizing and destabilizing forces associated with each specific residue type. Furthermore, comprehensive correlation analyses across all possible physicochemical transitions, alongside representative scatter plots for distinct biochemical environments, are presented in Figure S4, underscoring the model's robust generalizability.

Mutation sites are stratified into two distinct structural environments: the protein interior and the solvent‐exposed surface. This classification is determined by the relative accessible solvent area (rAsA), utilizing a threshold of 0.25. Residues satisfying rAsA<0.25 are categorized as interior, while those with rAsA≥0.25 are classified as surface. Biologically, mutations occurring within the protein interior generally exhibit a larger magnitude of destabilization compared to surface mutations. In our dataset, the average experimental ΔΔG for interior residues is −1.392 kcal/mol, compared to −0.522 kcal/mol for surface residues. This trend confirms the greater destabilizing effect in buried regions, attributable to the disruption of the tightly packed hydrophobic core essential for protein stability. The predictive performance of the model across these structural regions is illustrated in Figure 3c. The analysis reveals strong correlations in both environments: the interior region yields a PCC of 0.81 and an RMSE of 0.90 kcal/mol, while the surface region exhibits slightly superior performance with a PCC of 0.84 and an RMSE of 0.74 kcal/mol. This lower error on the surface likely reflects the lower variance in stability changes typical of solvent‐exposed sites. To visualize these structural definitions, Figure 3d depicts the structural shift in the protein 1ARR induced by the Q39G mutation. In this instance, residue 39 transitions from a Glutamine in the interior region of the wild type to a Glycine in the surface region of the mutant. By categorizing residues as either interior or surface for both wild‐type and mutant structures, we can examine the influence of continuous amino acid exposure on stability changes post‐mutation. Figure 3e displays the performance metrics across four specific mutation trajectories: Interior‐to‐Interior, Interior‐to‐Surface, Surface‐to‐Interior, and Surface‐to‐Surface. The model achieves robust performance across the majority of categories, with PCC values of 0.80 for Interior‐to‐Interior, 0.81 for Surface‐to‐Interior, and 0.82 for Surface‐to‐Surface. The slightly lower correlation observed for the Interior‐to‐Surface category with PCC value 0.74 is likely attributable to the significantly smaller sample size available for this specific mutation type.

### 2.3 Classification of mutation‐induced protein solubility changes

Changes in protein solubility arising from genetic mutations are critical determinants of protein stability and function, often leading to aggregation‐related diseases or altered biological activity. To model these effects, we utilized the dataset originally curated for PON‐Sol2 (Yang et al., 2021). This dataset comprises 6328 mutation samples derived from 77 distinct proteins. Mutations are categorized into three classes based on their impact on solubility: decrease, increase, and neutral (no change). The distribution and classification of mutation samples are illustrated in Figure S1. Specifically, the dataset contains 3136 samples demonstrating a decrease, 1026 samples indicating an increase, and 2166 samples showing no change. This distribution reveals a notable class imbalance with a ratio of approximately 1:0.69:0.33 (Decrease:Neutral:Increase), indicating a prevalence of mutations that reduce protein solubility. To establish a rigorous testing protocol and prevent data leakage, we strictly adopted the data partitioning methodology defined by the original PON‐Sol2 benchmark (Yang et al., 2021). Following the position‐stratified protocol, the resulting independent blind test set consisted of 662 variants comprising 338 solubility‐decreasing, 237 solubility‐increasing, and 87 neutral samples.

To rigorously assess the predictive capabilities of our framework, we implemented a dual validation strategy comprising a random 10‐fold cross‐validation and an independent blind test classification. In the cross‐validation phase, SheafLapNet was benchmarked against TopGBT (Wee et al., 2024), which is a model grounded in persistent homology, and the existing PON‐Sol2 predictive models (Yang et al., 2021), which employ feature selection techniques such as recursive feature elimination (RFE). To mitigate the effects of the inherent class imbalance, normalized accuracy scores were utilized as the primary metric for comprehensive performance assessment. Given that we are dealing with a K‐class problem with three distinct solubility classes, we rely on the Correct Prediction Ratio (CPR) and Generalized Squared Correlation (${\mathrm{GC}}^{2}$) as defined in Section S1.2 of the Supporting Information to provide a holistic assessment. Specifically, CPR measures the overall accuracy of the model while GC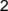 quantifies the correlation coefficient of the classification, ranging from 0 to 1. Larger values for these metrics denote better performance.

**FIGURE 4:**
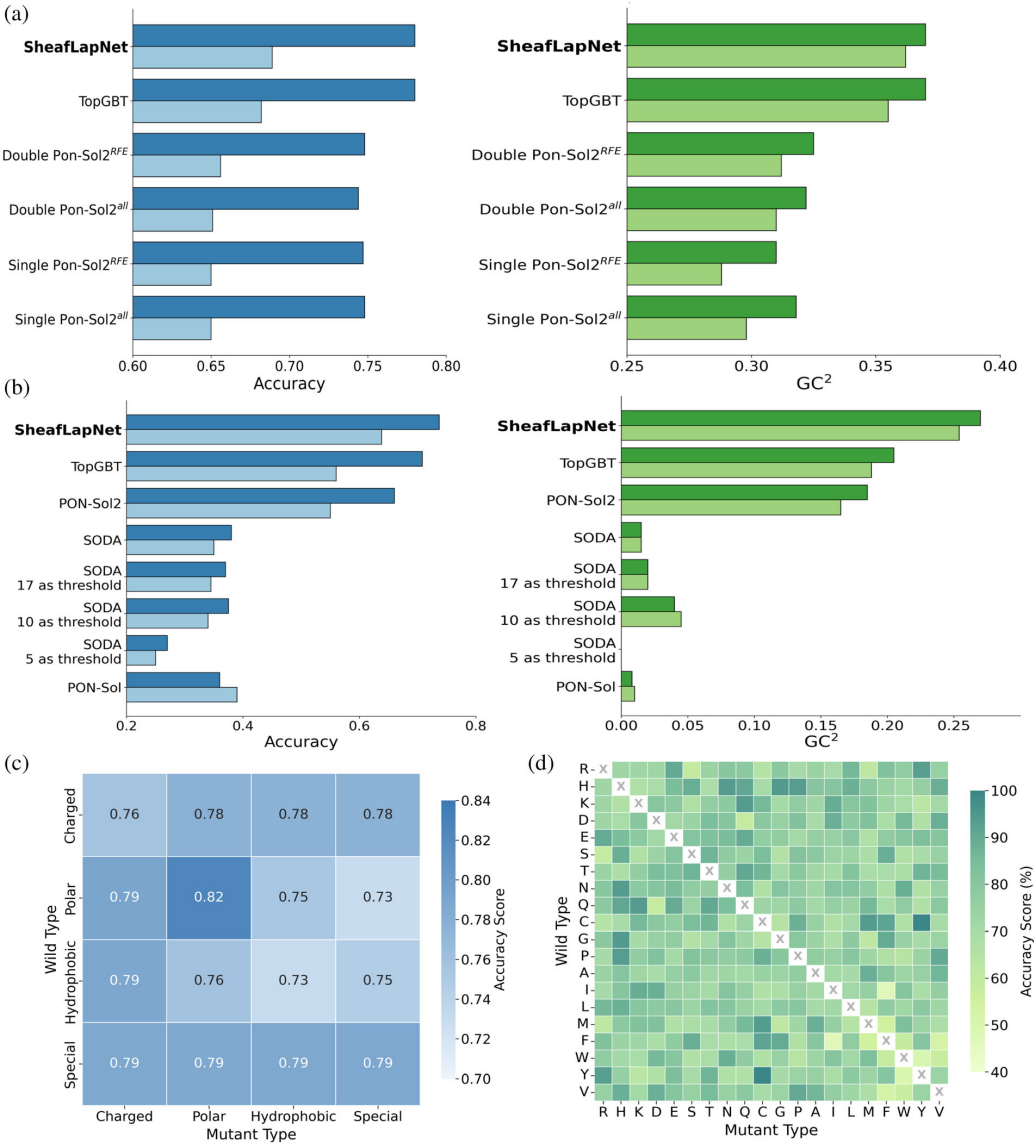
Illustration of model performance in classifying mutation‐induced protein solubility changes. Dark blue denotes Accuracy (CPR), light blue denotes Normalized Accuracy, Dark green denotes Generalized Squared Correlation (GC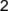), and light green denotes Normalized GC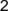. (a) Performance from 10‐fold cross‐validation on the PON‐Sol2 dataset, comparing SheafLapNet against existing models (Wee et al., 2024; Yang et al., 2021). For existing PON‐Sol2 models (Yang et al., 2021), RFE refers to recursive feature elimination, and all refers to the use of all features. (b) Independent blind test results on the PON‐Sol2 dataset, comparing SheafLapNet against state‐of‐the‐art models (Wee et al., 2024; Yang et al., 2016; Yang et al., 2021). (c) Model accuracy from 10‐fold cross‐validation stratified by physicochemical mutation groups. (d) Model accuracy from 10‐fold cross‐validation analyzed by specific amino acid substitution types, where X denotes samples with no mutation.

In the independent blind test setting, the proposed model, SheafLapNet, achieved normalized CPR and GC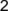 scores of 0.638 and 0.254, respectively. These results surpass the existing PON‐Sol2 models by up to 17.06% and 61.78%, and the persistent homology‐based TopGBT model by up to 13.52% and 38.04%. Given the multi‐class nature of the task, these metrics provide a comprehensive assessment of predictive performance, as illustrated in Figure 4b. A complete comparative evaluation of the CPR and GC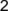 metrics against all baseline models is detailed in Table S7. The robustness of the Sheaf Laplacian framework was further confirmed through 10‐fold cross‐validation. In this evaluation, SheafLapNet maintained consistent predictive power, achieving a normalized CPR of 0.689 and a normalized GC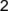 of 0.362. These scores exceed those of the PON‐Sol2 models by up to 5.03% and 16.03%, and TopGBT by up to 1.03% and 2.26%. Benchmark comparisons based on CPR and GC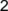 scores are presented in Figure 4a.

Switching focus to mutation types, our model's capability in classifying solubility changes also merits exploration across the 20 distinct amino acid types in the dataset. In addition to this, we subgroup amino acids as charged polar, hydrophobic, or special cases. Figure 4c displays accuracy scores for each mutation group pair, while Figure 4d shows scores for each amino acid pair. In this representation, the y‐axis labels the residue type of the original protein, whereas the x‐axis labels the residue type of the mutant. For a reverse mutation, the labels are taken with reverse solubility change unless the change is zero. The analysis reveals a clear disparity in predictive performance across mutation types. Notably, mutations transitioning from polar residues to polar groups achieve the highest classification accuracy. This is likely due to the direct correlation between surface polarity and solubility, resulting in predictable solvation energy changes that are effectively captured by the model's topological features. Conversely, transitions from polar to special residues or within the hydrophobic group exhibit comparatively lower performance. This reduced accuracy may reflect the challenge of modeling complex structural constraints, such as backbone rigidity introduced by special residues and the subtle packing interactions characteristic of the hydrophobic core.

## 3 MATERIALS AND METHODS

This section details the three distinct feature sets extracted from protein structures for our analysis: topological features derived from Persistent Sheaf Laplacians, physicochemical auxiliary descriptors, and evolutionary sequence embeddings. A comprehensive summary of the software packages utilized in this study is provided in Section S8 of the Supporting Information, while a detailed list of the auxiliary descriptors can be found in Section S7.

### 3.1 Persistent Sheaf Laplacian

Before introducing the Persistent Sheaf Laplacian, we first review the standard Persistent Laplacian. The construction begins by considering two simplicial complexes, K and L, such that K⊆L. We denote the simplicial chain complexes of K and L with real coefficients as $C^{K}$ and $C^{L}$, respectively. Since a chain group $C_{q}$ in a simplicial chain complex is formally generated by simplices, it naturally forms a finite‐dimensional inner product space, ensuring that the adjoint of the boundary map $\partial_{q}$ is well‐defined.

We define a subspace $C_{q+1}^{L,K}$ of $C_{q+1}^{L}$ as the set $\left\{c\in C_{q+1}^{L}|\partial_{q+1}^{L}\left(c\right)\in C_{q}^{K}\right\}$. Let $\partial_{q+1}^{L,K}$ denote the restriction of the boundary map $\partial_{q+1}^{L}$ to this subspace $C_{q+1}^{L,K}$. The q‐th Persistent Laplacian $\Delta_{q}^{L,K}$ is formally defined by the operator:

(1) $$\Delta_{q}^{L,K}=\partial_{q+1}^{L,K}{\left(\partial_{q+1}^{L,K}\right)}^{*}+{\left(\partial_{q}^{K}\right)}^{*}\partial_{q}^{K}.$$

To extend this framework to the Persistent Sheaf Laplacian, we must first define a cellular sheaf. A cellular sheaf S consists of a simplicial complex X (viewed as a cell complex) equipped with an assignment to each cell σ of X a finite‐dimensional vector space $S\left(\sigma \right)$, referred to as the stalk of S over σ. Additionally, for each face relation σ≼τ (where $\sigma \subset \bar{\tau }$), there exists a linear morphism of vector spaces denoted by $S_{\sigma ≼\tau }$, known as the restriction map. These maps satisfy the composition rule:

(2) $$S_{\rho ≼\tau }=S_{\sigma ≼\tau }S_{\rho ≼\sigma }$$

for any ρ≼σ≼τ, with $S_{\sigma ≼\sigma }$ being the identity map of $S\left(\sigma \right)$.

Analogous to a simplicial complex, a cellular sheaf generates a sheaf cochain complex. The q‐th sheaf cochain group $C_{S}^{q}$ is defined as the direct sum of stalks over q‐dimensional cells. By globally orienting the simplicial complex X to obtain a signed incidence relation $\left[\sigma :\tau \right]$ for each σ≼τ, the coboundary map $d^{q}:C_{S}^{q}\to C_{S}^{q+1}$ is defined by:

(3) $${\left.d^{q}\right|}_{S\left(\sigma \right)}=\sum_{\sigma ≼\tau }\left[\sigma :\tau \right]S_{\sigma ≼\tau }.$$

The Persistent Sheaf Laplacian is constructed by considering two cellular sheaves, S on K and T on L, such that K⊆L and the stalks and restriction maps of K are identical to those of L. The relationship between the cochain complexes of these sheaves and the persistent spectral structures is illustrated in the following commutative diagram:(4)

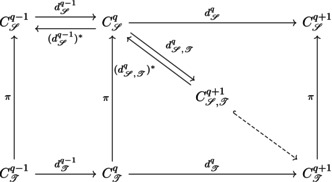




We define the subspace $C_{S,T}^{q+1}$ as $C_{S,T}^{q+1}=\left\{c\in C_{T}^{q+1}|{\left(d_{T}^{q}\right)}^{*}\left(c\right)\in C_{S}^{q}\right\}.$ Let $d_{S,T}^{q}$ denote the adjoint map of the restricted operator ${\left.{\left(d_{T}^{q}\right)}^{*}\right|}_{C_{S,T}^{q+1}}$. The q‐th PSL, denoted as $\Delta_{q}^{S,T}$, is defined as:

(5) $$\Delta_{q}^{S,T}={\left(d_{S,T}^{q}\right)}^{*}d_{S,T}^{q}+d_{S}^{q-1}{\left(d_{S}^{q-1}\right)}^{*}.$$

The spectral properties of the PSL provide key topological invariants. Specifically, the dimension of the zero‐eigenspace (kernel) of the operator corresponds to the q‐th persistent sheaf Betti number, denoted $\beta_{q}^{S,T}$ with $\beta_{q}^{S,T}=\dim\left(\ker\Delta_{q}^{S,T}\right)$.

This study utilizes cellular sheaves constructed on labeled simplicial complexes to model protein structures using atomic partial charges, following methodologies established in existing literature (Wei & Wei, 2025a). A general framework is first defined for constructing sheaves on a labeled simplicial complex X, where each vertex is associated with a quantity q. Let F:X→ℝ be a nowhere‐zero function. The sheaf is constructed by assigning the stalk ℝ to each simplex. For a face relation $\left[v_{0},\ldots ,v_{n}\right]\leq \left[v_{0},\ldots ,v_{n},v_{n+1},\ldots ,v_{m}\right]$ (where orientation is not relevant), the linear morphism $S\left(\left[v_{0},\ldots ,v_{n}\right]\leq \left[v_{0},\ldots ,v_{n},v_{n+1},\ldots ,v_{m}\right]\right)$ is defined as the scalar multiplication by:

(6) $$\frac{F\left(\left[v_{0},\ldots ,v_{n}\right]\right)q_{n+1}\cdots q_{m}}{F\left(\left[v_{0},\ldots ,v_{n},v_{n+1},\ldots ,v_{m}\right]\right)}.$$

To adapt this framework for protein analysis, non‐geometrical information is incorporated by employing atomic partial charges obtained from the PDB2PQR package (Jurrus et al., 2018). A Rips or Alpha filtration of graphs is constructed wherein vertices $v_{i}$ represent atoms and edges $e_{\mathrm{ij}}$ represent interactions between atoms $v_{i}$ and $v_{j}$. The cellular sheaf is defined such that each stalk is the real line ℝ. For the specific face relation $v_{i}≼e_{\mathrm{ij}}$ (where a vertex $v_{i}$ is a face of the edge $e_{\mathrm{ij}}$), the restriction morphism is explicitly defined as multiplication by $\frac{q_{j}}{r_{\mathrm{ij}}}$, where $q_{j}$ is the partial charge of the neighboring atom $v_{j}$, and $r_{\mathrm{ij}}$ represents the Euclidean length of the edge $e_{\mathrm{ij}}$. The harmonic spectra of the resulting PSL reveal topological invariants, while the nonharmonic spectra represent geometric information of the data. Consequently, these spectra are utilized as the input features for the SheafLapNet model.

**FIGURE 5:**
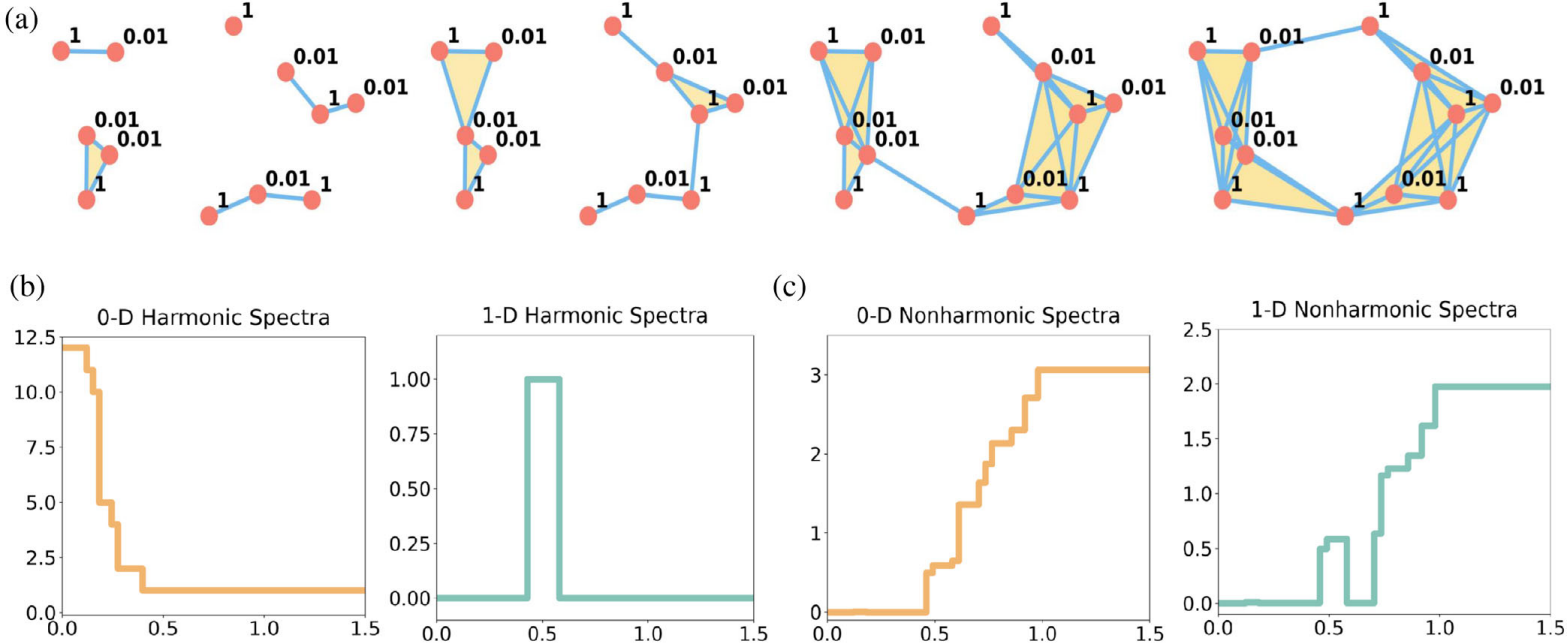
Illustration of persistent sheaf Laplacians. (a) A filtration process of the Rips complex from point cloud data. (b) The persistent multiplicity of zero eigenvalues from the sheaf Laplacian matrices in 0‐D and 1‐D dimensions. (c) The minimal value of non‐zero eigenvalues from the zero‐dimensional and one‐dimensional sheaf Laplacian matrices.

To illustrate the Persistent Sheaf Laplacian framework, we present an example using a point cloud dataset consisting of 12 points, as depicted in Figure 5. The topological structure is modeled through a Vietoris–Rips (VR) complex filtration, visualized in Figure 5a. Here, heterogeneous information is explicitly mapped onto the domain, with nodes assigned distinct “charge” values of 1 and 0.01. As the filtration parameter increases, the connectivity of the point cloud evolves, generating a sequence of nested simplicial complexes that serve as the domain for our sheaf‐theoretic analysis. Figure 5b presents the corresponding harmonic spectral analysis derived from the persistent sheaf Laplacians, specifically plotting the persistent multiplicities of the zero eigenvalues. These multiplicities correspond to the Sheaf Betti numbers, which generalize standard topological invariants by incorporating heterogeneous information such as atomic partial charges. Complementing this harmonic analysis, Figure 5c displays the non‐harmonic spectral information, specifically the evolution of the minimum non‐zero eigenvalues. These values quantify the connectivity strength and local geometric rigidity, offering a continuous measure of the intrinsic physical and chemical interactions encoded within the sheaf.

### 3.2 Sheaf Laplacian feature generation for protein

In the SheafLapNet architecture, protein structures are modeled as sets of simplicial complexes, from which PSLs are computed to extract topological features. To balance computational efficiency with the capability to capture essential physical interactions, the model employs an element‐specific and site‐specific atom subsetting strategy. Atoms within the three‐dimensional protein structure are partitioned into mutation‐site atoms, denoted as $A_{m}$, and mutation neighborhood atoms located within a cutoff radius r, denoted as $A_{\mathrm{mn}}\left(r\right)$. This partitioning is applied to both wild‐type and mutant structures. Leveraging the element‐specific representation framework (Cang & Wei, 2018), the model focuses on interactions involving carbon (C), nitrogen (N), and oxygen (O) atoms. By considering the intersections of these element types between the mutation site and its neighborhood, nine distinct pairwise atom combinations are constructed. These combinations encode specific interaction types. For instance, the pairing of carbon atoms in the mutation site ($A_{C}\cap A_{m}$) with those in the neighborhood ($A_{C}\cap A_{\mathrm{mn}}\left(r\right)$) captures hydrophobic C–C interactions, whereas combinations involving carbon and oxygen ($A_{C}\cap A_{m}$ and $A_{O}\cap A_{\mathrm{mn}}\left(r\right)$) characterize polar C–O interactions. These element‐specific and site‐specific sets subsequently underpin the multiscale sheaf Laplacian embeddings.

To accurately capture the topological interactions within the defined atom sets, we construct VR complexes (Vietoris, 1927) and Alpha complexes (Edelsbrunner, 2011). For the VR complex construction, we employ a modified distance function, d, designed to specifically isolate interfacial interactions between distinct atom sets (e.g., between the mutation site $A_{m}$ and the neighborhood $A_{\mathrm{mn}}\left(r\right)$). The modified metric is defined as:

(7) $$d\left(a_{i},a_{j}\right)=(\begin{array}{ll} E_{d}\left(a_{i},a_{j}\right) & \text{if}\left(a_{i}\in A_{m}\wedge a_{j}\in A_{\mathrm{mn}}\left(r\right)\right)\vee \left(a_{i}\in A_{\mathrm{mn}}\left(r\right)\wedge a_{j}\in A_{m}\right),\\ \infty & \text{otherwise, } \end{array}$$

where $E_{d}\left(a_{i},a_{j}\right)$ denotes the Euclidean distance between atoms $a_{i}$ and $a_{j}$. By assigning an infinite distance to atom pairs belonging to the same set, this metric effectively filters out intra‐set edges, thereby focusing the topological analysis exclusively on the interactions between the mutation site and its surrounding environment. Complementing this, Alpha complexes (Edelsbrunner, 2011) are constructed using the standard Euclidean distance on the same atom sets to capture local geometric constraints.

**FIGURE 6:**
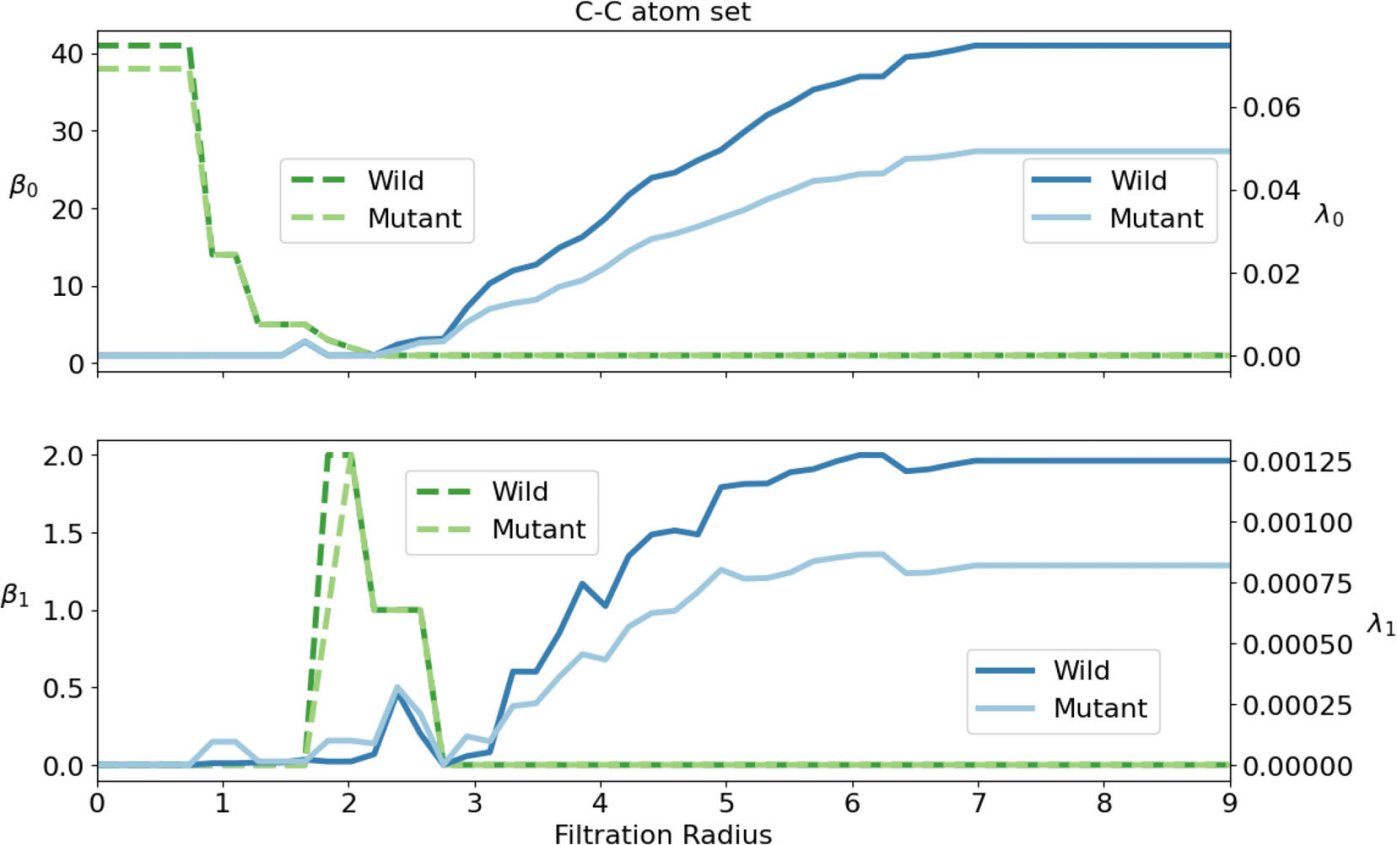
An illustration of PSL descriptors for C–C interactions from VR complexes using modified distance filtration at residue 37 mutation site from L to S in protein 1A5E of S2648 dataset. Top panel: Illustration of zero‐dimensional PSL descriptors. Bottom panel: Illustration of one‐dimensional PSL descriptors. The left axis and dashed green lines represent the Sheaf Betti numbers $\beta_{0}$ and $\beta_{1}$. The right axis and solid blue lines represent the minimal nonzero eigenvalues $\lambda_{0}$ and $\lambda_{1}$ of the persistent sheaf Laplacian.

**FIGURE 7:**
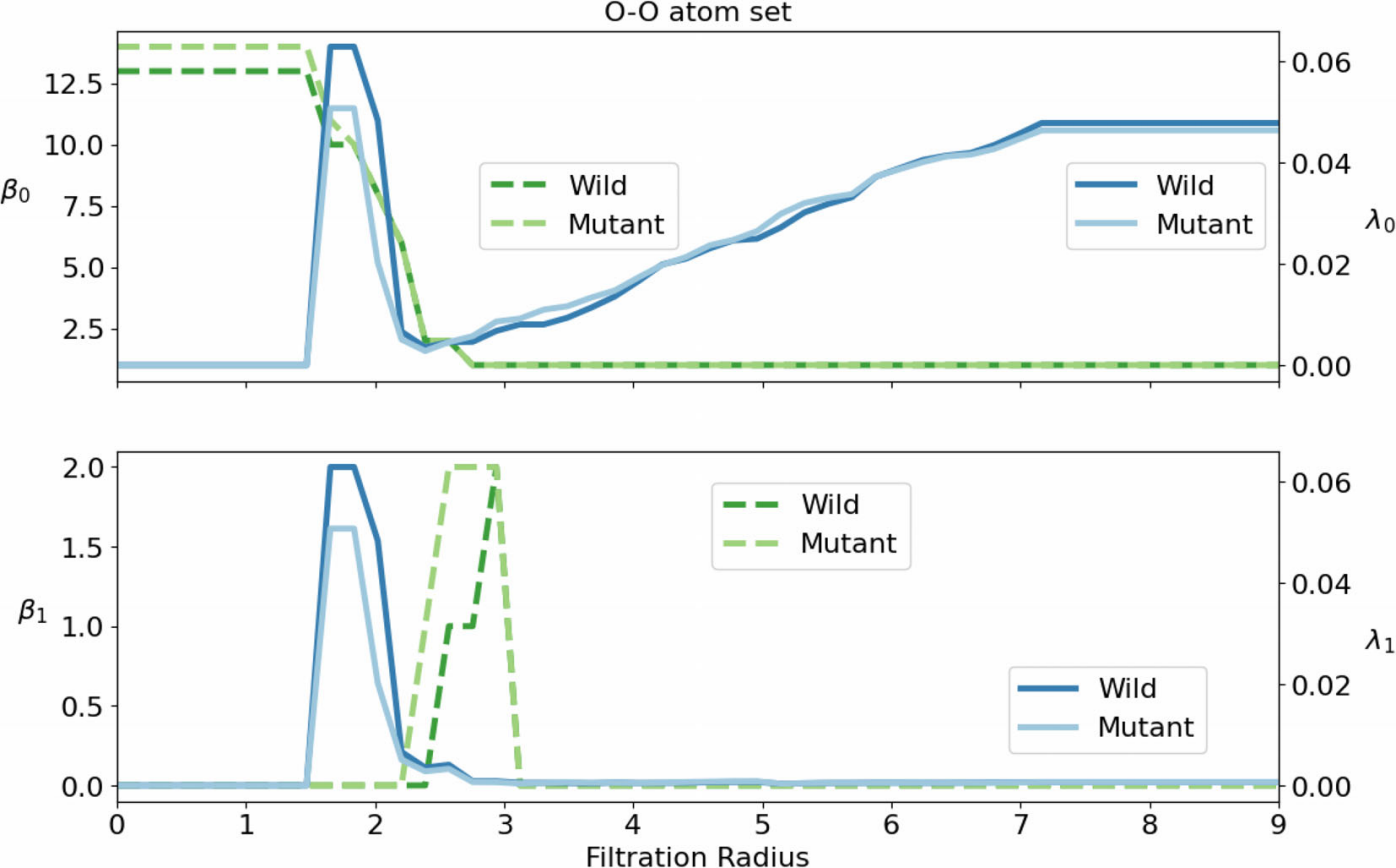
An illustration of PSL descriptors for O–O interactions from VR complexes using modified distance filtration at residue 37 mutation site from L to S in protein 1A5E of S2648 dataset. Top panel: Illustration of zero‐dimensional PSL descriptors. Bottom panel: Illustration of one‐dimensional PSL descriptors. The left axis and dashed green lines represent the Sheaf Betti numbers $\beta_{0}$ and $\beta_{1}$. The right axis and solid blue lines represent the minimal nonzero eigenvalues $\lambda_{0}$ and $\lambda_{1}$ of the persistent sheaf Laplacian.

Spectral features are extracted from the constructed complexes to serve as local PSL descriptors. In this study, a cutoff distance of 16Å from the mutation site is employed to identify mutation neighborhood atoms. For the VR complex, the filtration is conducted over a range of 3Å to 9Å with a step size of 1Å. For each filtration step, spectral properties are derived from the Persistent Sheaf Laplacian. Regarding the harmonic components, the count of zero eigenvalues is recorded, generating a 7‐dimensional feature vector for each atom set. For the non‐harmonic components, eight statistical properties are extracted from the non‐zero eigenvalue spectrum: maximum, minimum, mean, sum, standard deviation, variance, and the eigenvalue count. This procedure yields a 63‐dimensional feature vector per atom set. Similarly, for the Alpha complex, the one‐dimensional harmonic and non‐harmonic components of the persistent sheaf Laplacians are analyzed using the same statistical extraction method. The final topological feature representation is constructed by combining the extracted features from the zero‐dimensional VR models and the one‐dimensional Alpha models. These feature vectors are computed for the wild‐type structure, the mutant structure, and the difference between them. The concatenation of these vectors results in a comprehensive feature representation for a single protein, characterized by a 3402‐dimensional vector. To illustrate the discriminative capacity of these features, Figures 6 and 7 depict the zero‐dimensional and one‐dimensional PSL descriptors for both wild‐type and mutant structures of protein 1A5E from the S2648 dataset. Figure 6 utilizes atom sets $A_{C}\cap A_{m}$ and $A_{C}\cap A_{\mathrm{mn}}\left(r\right)$ to generate VR complexes with a modified distance d‐based filtration, effectively revealing hydrophobic C–C interactions. Similarly, Figure 7 utilizes atom sets $A_{O}\cap A_{m}$ and $A_{O}\cap A_{\mathrm{mn}}\left(r\right)$ to generate VR complexes with d‐based filtration, revealing the O–O interactions.

### 3.3 Auxiliary features

In addition to topological features, our model integrates a comprehensive set of physicochemical descriptors at both the atom and residue levels to characterize the mutation environment. At the atom level, we compute solvent‐excluded surface areas, partial atomic charges, Coulombic and van der Waals interaction energies, and electrostatic solvation free energies (Chen et al., 2011; Jurrus et al., 2018; Rocchia et al., 2001). Here, electrostatic solvation free energies were computed using the MIBPB solver for the Poisson–Boltzmann model (Chen et al., 2011). At the residue level, the feature set comprises the amino acid composition and physical properties of the local neighborhood, mutation‐induced pKa shifts, evolutionary conservation scores derived from Position‐Specific Scoring Matrices (PSSM), and predicted secondary structure parameters. Detailed definitions and calculation protocols are provided in Section S3 of the Supporting Information.

### 3.4 Sequence features

Protein large language models (pLLMs), such as ESM (Evolutionary Scale Modeling) (Rives et al., 2021) and ProtTrans (Elnaggar et al., 2021), have demonstrated remarkable efficacy in capturing deep evolutionary patterns from hundreds of millions of unannotated sequences. To incorporate this rich evolutionary context into our algebraic framework, we employ the ESM‐2 Transformer architecture (Lin et al., 2023). Trained via self‐supervised masked language modeling (MLM) on a comprehensive protein sequence database, this architecture comprises 33 Transformer layers and approximately 650 million parameters. For a given input sequence, the model produces contextualized token embeddings of dimension 1280. We extract these representations from the final layer and compute the mean across the sequence length, yielding a single fixed‐length 1280‐dimensional vector. To explicitly capture the impact of genetic variations, we generate these embeddings for both the wild‐type and mutant sequences and their element‐wise difference. These are subsequently concatenated to form a final 3840‐dimensional evolutionary feature vector used in our predictive model. The optimality of this specific global concatenation strategy over alternative localized extraction methods is demonstrated in Table S3. Furthermore, an ablation study comparing the standard ESM‐2 embeddings against the advanced ESM‐C architecture is provided in Table S4, confirming the framework's adaptability and consistent predictive robustness.

## 4 CONCLUSION

Accurately predicting the effects of genetic mutations on protein stability and solubility is fundamental to understanding the molecular basis of disease and advancing precision medicine. However, experimental characterization of these widely occurring variants is resource‐intensive, and existing computational models often suffer from limited interpretability or treat stability and solubility as disjoint tasks. Therefore, developing unified, rigorous predictive frameworks capable of capturing intrinsic molecular interactions is a critical imperative. To address this challenge, we introduced SheafLapNet, a novel Topological deep learning framework that leverages the persistent sheaf Laplacian to predict mutation‐induced changes in both protein stability and solubility. By synergizing the heterogeneous geometric information encoded by the persistent sheaf Laplacian with auxiliary physicochemical descriptors and evolutionary sequence embeddings from the pre‐trained ESM‐2 transformer, our model achieves a mathematically grounded and robust representation of molecular alterations. This integrative approach captures the multiscale and mechanistic nuances of mutation impacts, resulting in state‐of‐the‐art predictive performance across comprehensive benchmark datasets, including S2648, S350, PON‐Sol2, and S669. Crucially, to ensure true generalizability, we rigorously evaluated SheafLapNet under stringent, homology‐free conditions (enforcing <25% sequence identity) using the independent S669 blind test set. The model's sustained accuracy and near‐perfect thermodynamic anti‐symmetry under these strict controls definitively validate its robust predictive capabilities across highly divergent protein families.

Moving forward, our framework can be extended through both biophysical and mathematical refinements. From a biophysical perspective, a critical frontier remains the explicit structural modeling of the unfolded state ensemble (Lee et al., 2026). Future iterations will aim to explicitly simulate and topologically analyze this highly dynamic state. Incorporating such representations alongside the native folded structure will provide a more comprehensive geometric formulation of the thermodynamic folding cycle. Mathematically, the rapidly evolving field of topological data analysis offers additional opportunities for refinement through distinct mathematical invariants, such as the persistent Path Laplacian (Wang & Wei, 2023), persistent hyperdigraph Laplacians (Chen et al., 2023), the persistent Dirac operator (Ameneyro et al., 2024; Suwayyid & Wei, 2024), and sheaf or copresheaf neural networks (Barbero et al., 2022; Hajij et al., 2025). These advanced tools may provide deeper insights into the directed and higher‐order interactions within protein structures that standard simplicial complexes may not fully capture, potentially enabling further improvements in predictive accuracy and interpretability. In addition, future studies may further examine the complementarity of persistent sheaf Laplacian representations with emerging structure‐aware protein foundation models, such as ESM3 and ProstT5, through controlled benchmarking studies that standardize input modalities, embedding extraction protocols, downstream training procedures, and evaluation datasets.

## AUTHOR CONTRIBUTIONS

**Grace Qian:** Writing – review and editing. **Xi Chen:** Writing – review and editing. **Junjie Wee:** Software; methodology; writing – review and editing. **Yiming Ren:** Visualization; writing – review and editing; writing – original draft; software. **Guo‐Wei Wei:** Writing – review and editing; supervision; conceptualization; methodology.

## CONFLICT OF INTEREST STATEMENT

The authors declare no conflicts of interest.

## Supporting information


**Table S1.** SheafLapNet model hyperparameters.
**Table S2**. Feature ablation study evaluated on the S2648‐based stability benchmark for regression and the PON‐Sol2 blind test set for classification. PSL stands for Persistent Sheaf Laplacian, Phy denotes Physicochemical, and Seq represents Sequence utilizing ESM‐2.
**Table S3**. Ablation of sequence embedding pooling strategies on the S2648 blind test. All models utilize ESM‐2 alongside Phy and PSL features. Both Residue‐specific Embedding and Mean Embedding strategies incorporate the WT sequence, MT sequence, and their element‐wise difference.
**Table S4**. Ablation of pretrained sequence embedding models on the S2648 stability benchmark. The sequence pooling strategy is fixed to the mean embedding of WT and MT sequences with their element‐wise difference.
**Table S5**. Comparative benchmark evaluating the performance of various methods on the S669 independent blind test set. Results for baseline methods were collected from the corresponding publications or reported benchmark studies, while SheafLapNet results were obtained in this work using S2648 as the training set. Blank entries indicate that the corresponding metrics were not reported in the original source.
**Table S6**. Comparison of SheafLapNet's predictive performance with the reported PCC and RMSE for the mutation‐induced protein stability change prediction datasets. Existing results are referenced from Benevenuta et al. (2021) unless otherwise stated.^*a*^ Results obtained from Karczewski et al. (2020).^*b*^ Results obtained from Gong et al. (2023).^*c*^ According to reference Gong et al. (2023) the data from the online server has PCC (RMSE) of 0.59 (1.28) and 0.70 (1.13) for INPS and mCSM respectively in the task of S350 set. The *n* column denotes the number of mutation samples successfully processed by each method.
**Table S7**. Performance of SheafLapNet compared with existing models on the PON‐Sol2 independent blind test classification for solubility prediction. Except for SheafLapNet, performance metrics for all baseline methods were retrieved from Jurrus et al. (2018).
**Table S8**. Summary of specific training and evaluation datasets, alongside the required computational execution time, for each stability and solubility prediction task. Time is reported in minutes and encompasses the complete training and prediction cycle across all ten independently seeded models.
**Figure S1**. Distribution of mutation samples in the PON‐Sol2 dataset. (a) Overall categorization of mutations based on their impact on solubility: decrease, neutral, or increase. (b) Stratification of sample counts across the training and blind test sets.
**Figure S2**. Illustration of model performance on the S350 and S2648 datasets in protein stability changes upon mutation. (a). 5‐fold cross‐validation performance of SheafLapNet for S2648 dataset compared to existing state‐of‐the‐art models (Benevenuta et al., 2021; Gong et al., 2023; Karczewski et al., 2020). (b). Blind test performance of SheafLapNet for S350 dataset compared to existing state‐of‐the‐art models (Benevenuta et al., 2021; Cang et al., 2018; Gong et al., 2023; Karczewski et al., 2020). (c). Comparison of experimental protein stability changes with predicted ones from SheafLapNet for the S2648 dataset. (d). Comparison of experimental protein stability changes with predicted ones from SheafLapNet for S350 dataset. Note: An average of 99.58% of the validation mutations in the 5‐fold cross‐validation (a, c) and 346 out of 350 mutations in the blind test (b, d) share >25% sequence identity with their respective training sets.
**Figure S3**. Distribution of mutation samples and corresponding MSE across amino acid substitutions in the S2648 dataset. The left panel displays the sample count for each specific substitution pair, where the x‐axis corresponds to the wild‐type residue and the y‐axis denotes the mutant residue. The right panel illustrates the MSE for these identical substitution pairs to highlight the predictive strengths and vulnerabilities of the model. In both panels, an X indicates the deliberate exclusion or absence of mutation samples for that specific transition.
**Figure S4**. The correlation analysis of predicted versus experimental protein stability changes. The top panel presents a heatmap detailing the PCC across all possible physicochemical transitions between wild‐type and mutated residues. The white cell marked with an X indicates the deliberate exclusion of synonymous Alanine to Alanine mutations. The bottom panel provides scatter plots for six representative mutation pairs to illustrate predictive accuracy and error distribution across diverse biochemical environments.

## Data Availability

The data and code in this study can be found in https://github.com/yren24/SheafLapNet/tree/main.
