# Supplementary material for: Persistent sheaf Laplacian analysis of protein stability and solubility changes upon mutation
Source: Protein Sci. 2026 Jul 10;35(8):e70700. doi: 10.1002/pro.70700 (PMC13351944; doi:10.1002/pro.70700)
Supplement: Supplementary file 1 — Table S1. SheafLapNet model hyperparameters. Table S2. Feature ablation study evaluated on the S2648‐based stability benchmark for regression and the PON‐Sol2 blind test set for classification. PSL stands for Persistent Sheaf Laplacian, Phy denotes Physicochemical, and Seq represents Sequence utilizing ESM‐2. Table S3. Ablation of sequence embedding pooling strategies on the S2648 blind test. All models utilize ESM‐2 alongside Phy and PSL features. Both Residue‐specific Embedding and Mean Embedding strategies incorporate the WT sequence, MT sequence, and their element‐wise difference. Table S4. Ablation of pretrained sequence embedding models on the S2648 stability benchmark. The sequence pooling strategy is fixed to the mean embedding of WT and MT sequences with their element‐wise difference. Table S5. Comparative benchmark evaluating the performance of various methods on the S669 independent blind test set. Results for baseline methods were collected from the corresponding publications or reported benchmark studies, while SheafLapNet results were obtained in this work using S2648 as the training set. Blank entries indicate that the corresponding metrics were not reported in the original source. Table S6. Comparison of SheafLapNet's predictive performance with the reported PCC and RMSE for the mutation‐induced protein stability change prediction datasets. Existing results are referenced from Benevenuta et al. (2021) unless otherwise stated. a Results obtained from Karczewski et al. (2020). b Results obtained from Gong et al. (2023). c According to reference Gong et al. (2023) the data from the online server has PCC (RMSE) of 0.59 (1.28) and 0.70 (1.13) for INPS and mCSM respectively in the task of S350 set. The n column denotes the number of mutation samples successfully processed by each method. Table S7. Performance of SheafLapNet compared with existing models on the PON‐Sol2 independent blind test classification for solubility prediction. Except for SheafLapNet, [file PRO-35-e70700-s001.pdf]

Supplementary Information for

# Persistent Sheaf Laplacian Analysis of Protein Stability and Solubility Changes upon Mutation

Yiming Ren[1], Junjie Wee[1], Xi Chen[2], Grace Qian[3], and Guo-Wei Wei[1,4,5*]

[1]Department of Mathematics,
Michigan State University, East Lansing, MI 48824, USA.
[2]The Frazer School, 4700 NW 89 Blvd, Gainesville, FL 32606, USA
[3]Lassiter High School, Marietta, GA 30066, USA
[4]Department of Mathematics,
University of Georgia, Athens, GA 30602, USA.
[5]Department of Biochemistry and Molecular Biology,
University of Georgia, Athens, GA 30602, USA.

*Corresponding author. Email: guowei.wei@uga.edu

# S1 Evaluation Metrics

## S1.1 Regression Metrics for Stability Prediction

For the mutation-induced protein stability datasets (S2648 and S350), we employed the Pearson correlation coefficient (PCC) and the root mean squared error (RMSE) to quantify predictive performance.

Let $N$ be the total number of samples. For the $i$-th sample, let $y_i$ denote the ground truth experimental value and $\hat{y}_i$ denote the predicted value. The mean of the ground truth values is denoted by $\bar{y}$, and the mean of the predicted values is denoted by $\bar{\hat{y}}$. The PCC is defined as:

$$\mathrm{PCC} = \frac{\sum_{i=1}^{N}(y_i - \bar{y})(\hat{y}_i - \bar{\hat{y}})}{\sqrt{\sum_{i=1}^{N}(y_i - \bar{y})^2}\sqrt{\sum_{i=1}^{N}(\hat{y}_i - \bar{\hat{y}})^2}}. \tag{S1}$$

The RMSE is calculated as:

$$\mathrm{RMSE} = \sqrt{\frac{1}{N}\sum_{i=1}^{N}(y_i - \hat{y}_i)^2}. \tag{S2}$$

To evaluate the thermodynamic consistency of the predictive models on the independent S669 blind test set, we further introduce the anti-symmetry correlation ($r_{f-r}$) and the anti-symmetry bias ($\langle\delta\rangle$). Let $\hat{y}_i^{(fwd)}$ denote the predicted stability change for the forward (direct) mutation, and $\hat{y}_i^{(rev)}$ denote the predicted stability change for the corresponding reverse mutation. The anti-symmetry correlation, $r_{f-r}$, evaluates the linear relationship between the direct and inverse predictions and is defined as the Pearson correlation between them:

$$r_{f-r} = \mathrm{PCC}(\hat{y}^{(fwd)}, \hat{y}^{(rev)}). \tag{S3}$$

A perfectly anti-symmetric and thermodynamically consistent model yields an ideal correlation of $r_{f-r} = -1$. The anti-symmetry bias, $\langle\delta\rangle$, quantifies the systematic deviation from perfect thermodynamic reversibility (where $\Delta\Delta G_{fwd} = -\Delta\Delta G_{rev}$). It is defined as:

$$\langle\delta\rangle = \frac{1}{2N}\sum_{i=1}^{N}\left(\hat{y}_i^{(fwd)} + \hat{y}_i^{(rev)}\right). \tag{S4}$$

An ideal model perfectly captures the bidirectional thermodynamic laws governing protein stability, resulting in an anti-symmetry bias of $\langle\delta\rangle = 0$.

## S1.2 Classification Metrics for Solubility Prediction

To evaluate the performance of SheafLapNet on the mutation-induced protein solubility dataset (PON-Sol2), which involves a multi-class prediction problem categorizing mutations as increased, decreased, or neutral, we utilized two primary metrics: Accuracy and the Generalized Squared Correlation ($\mathrm{GC}^2$).

Let $K$ denote the number of classes and $N$ the total number of samples. We define $z_{ij}$ as the count of samples belonging to the true class $i$ that are predicted as class $j$. Consequently, the marginal sums are given by $x_i = \sum_{j=1}^{K} z_{ij}$ (total samples in true class $i$) and $y_j = \sum_{i=1}^{K} z_{ij}$ (total samples predicted as class $j$). The Accuracy, also known as the correct prediction ratio (CPR), is defined as:

$$\mathrm{Accuracy} = \frac{1}{N}\sum_{i=1}^{K} z_{ii}. \tag{S5}$$

The Generalized Squared Correlation ($\mathrm{GC}^2$) provides a measure of correlation that accounts for the distribution of classes. Assuming independence between the true and predicted labels,

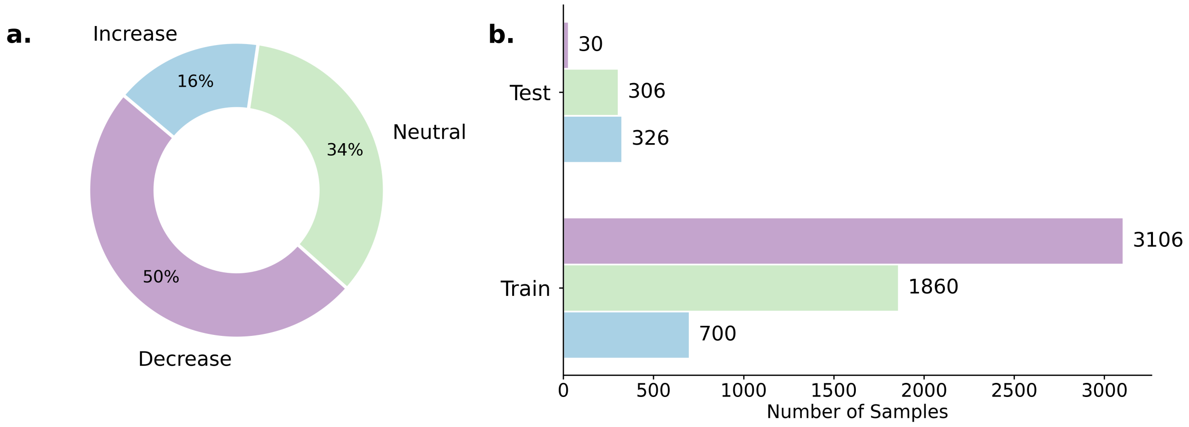


Figure S1: Distribution of mutation samples in the PON-Sol2 dataset. (a) Overall categorization of mutations based on their impact on solubility: decrease, neutral, or increase. (b) Stratification of sample counts across the training and blind test sets.

the expected frequency for the $(i, j)$-th entry of the confusion matrix is $e_{ij} = \frac{x_i y_j}{N}$. The $\mathrm{GC}^2$ is then calculated as:

$$GC^2 = \frac{1}{N(K-1)} \sum_{i=1}^{K} \sum_{j=1}^{K} \frac{(z_{ij} - e_{ij})^2}{e_{ij}}. \tag{S6}$$

Given the inherent class imbalance in the solubility datasets, normalization strategies were applied where appropriate to ensure the reliability of these metric calculations.

## S2 Dataset distribution

As outlined in the main text, the S2648 and PON-Sol2 datasets provide stringent benchmarks for evaluating model robustness under distinct distributional challenges. The S2648 dataset facilitates the analysis of direct amino acid substitutions; however, as detailed in Figure S3, the distribution of mutant samples reveals a highly non-uniform sampling density. In contrast, the PON-Sol2 dataset was developed to support machine learning-based prediction of mutation-induced solubility changes. Comprising both experimentally validated and literature-curated variants, it is widely used for multi-class classification tasks. The breakdown of mutation samples by solubility effect is summarized in Figure S1, which illustrates the significant class imbalance characterizing this dataset.

## S3 SheafLapNet Model Architecture and Hyperparameters

The SheafLapNet framework employs a deep neural network designed to efficiently process high-dimensional concatenated vectors comprising extracted Persistent Sheaf Laplacian features, Transformer-based sequence embeddings, and handcrafted physicochemical auxiliary vectors. The core network architecture features a feed-forward multi-layer perceptron consisting of six fully connected hidden layers with a decreasing neuron configuration of 2048, 1024, 1024, 512, 512, and 64 units. To introduce non-linearity and enhance the representational capacity of the model, ReLu activations are applied immediately following each hidden layer. The final output layer is inherently task-dependent, formulated to generate either a continuous scalar value for stability prediction regression tasks or discrete class probabilities for solubility prediction classification tasks.

Model optimization is driven by the AdamW optimizer with a designated weight decay of 0.05 to mitigate overfitting. Furthermore, the learning rate is dynamically modulated through-

out the training process using a OneCycleLR scheduler, configured with a thirty percent warm-up phase, to ensure stable and accelerated convergence. The objective function is also tailored to the specific downstream task, utilizing an L1 Loss function for regression and a Cross-Entropy Loss function for classification.

Given the fundamental differences in dataset scale and task objectives, the training hyperparameters were distinctly optimized for stability and solubility predictions. For the regression-based protein stability datasets, training was conducted over 50 epochs utilizing a batch size of 64 and a maximum learning rate of 0.0002. Conversely, for the classification-based protein solubility datasets, the model achieved optimal convergence over 20 epochs with a larger batch size of 128 and a maximum learning rate of 0.001. To ensure robust generalization and mitigate variance arising from network initialization, the final predictive output for any given mutation is computed as the average of predictions generated by ten independently trained models, each initialized with a distinct random seed. A comprehensive summary of the architectural configurations and training hyperparameters is provided in Table S1.

Table S1: SheafLapNet model hyperparameters.

| Hyperparameter | Stability Prediction | Solubility Prediction |
|---|---|---|
| Task Type | Regression | Classification |
| Hidden Layers Configurations | 2048, 1024, 1024, 512, 512, 64 | |
| Activation Function | ReLu | |
| Optimizer | AdamW | |
| Weight Decay | 0.05 | |
| Learning Rate Scheduler | OneCycleLR | |
| Warm-up Phase | 30 Percent | |
| Loss Function | L1 Loss | Cross-Entropy Loss |
| Maximum Learning Rate | 0.0002 | 0.001 |
| Batch Size | 64 | 128 |
| Training Epochs | 50 | 20 |

# S4 Ablation Studies and Feature Analysis

## S4.1 Performance Comparison of Different Feature Modalities

In our SheafLapNet model, we incorporate three types of features: topological features derived from persistent sheaf Laplacians, sequence features obtained from a pretrained Transformer model, and physicochemical features. To evaluate the contribution of each feature type, we construct several ablation models using different combinations of these features. We denote the topological, sequence, and physicochemical features as PSL, Seq, and Phy, respectively. We evaluated these variations on both the S2648-based stability evaluation, a regression task for predicting stability changes denoted as $\Delta\Delta G$, and the PON-Sol2 blind test set, a classification task for predicting solubility variations.

To rigorously quantify the impact of the topological embeddings, we trained a variant of the model stripped of the PSL features, relying exclusively on the sequence and physicochemical features. As demonstrated in Table S2, the removal of the PSL-derived features yields a distinct and consistent performance gap. On the S2648 test set, omitting PSL caused the PCC to decrease from 0.82 to 0.80, while the RMSE increased from 0.90 to 0.93. This degradation is profoundly evident on the independent PON-Sol2 classification set, where the Normalized Accuracy dropped from 0.638 to 0.597 and the $GC^2$ fell significantly from 0.270 to 0.227 without the topological features. Such a consistent drop across multiple metrics suggests that the persistent

sheaf Laplacian provides structural and geometric insights that are not natively encoded within the sequence embeddings.

We also investigated the specific contributions of the physicochemical features to the overall predictive robustness of the framework. To isolate this effect, we evaluated a reduced architectural variant that relied entirely on the advanced sequence embeddings and topological representations across both the S2648 stability and PON-Sol2 solubility blind test sets, as shown in Table S2. The omission of these explicit biophysical properties resulted in a quantifiable degradation of predictive accuracy, evidenced on the S2648 dataset by a reduction in the PCC to 0.81 and a concurrent increase in the RMSE to 0.93. This loss of performance was similarly pronounced during the classification of solubility variations within the PON-Sol2 test set, where the Normalized Accuracy experienced a notable drop to 0.599 when compared against the fully integrated model. Moreover, the optimal and most reliable performance of the SheafLapNet architecture remains fundamentally dependent on the synergistic integration of all three distinct feature modalities.

Table S2: Feature ablation study evaluated on the S2648-based stability benchmark for regression and the PON-Sol2 blind test set for classification. PSL stands for Persistent Sheaf Laplacian, Phy denotes Physicochemical, and Seq represents Sequence utilizing ESM-2.

| | S2648 | | PON-Sol2 | |
|---|---|---|---|---|
| Features | PCC | RMSE | Norm. Acc. | $GC^2$ |
| Seq + Phy + PSL | 0.82 | 0.90 | 0.638 | 0.270 |
| Seq + PSL | 0.81 | 0.93 | 0.599 | 0.225 |
| Seq + Phy | 0.80 | 0.93 | 0.597 | 0.227 |
| Seq | 0.76 | 1.02 | 0.588 | 0.207 |
| Phy | 0.78 | 0.99 | 0.497 | 0.109 |
| PSL | 0.73 | 1.08 | 0.496 | 0.107 |

## S4.2 Analysis of Sequence Representation Strategies

Having established the necessity of all three feature domains, we further analyzed how different extraction methodologies for the sequence embeddings influence the model's predictive behavior. A fundamental question in utilizing full-length protein sequence embeddings is whether the global contextual mean dilutes the highly localized signal of a single-point mutation. To investigate this, we evaluated several pooling and concatenation strategies on the S2648 dataset, with results detailed in Table S3. Our primary methodology concatenates the global mean embeddings of both the wild-type (WT) and mutated (MT) sequences, and their element-wise difference. The analysis reveals that relying exclusively on either the WT or the MT global mean embedding leads to a measurable loss of predictive accuracy, increasing the RMSE to 0.97. This degradation highlights that the network's predictive capacity depends heavily on capturing the explicit contrastive difference between the two sequence states. Furthermore, substituting the global mean embeddings with strictly localized, residue-specific embeddings comprising the residue-level WT, MT, and their difference at the precise mutation site yielded identical predictive performance (PCC of 0.82 and RMSE of 0.90). This equivalence demonstrates that the proposed feature integration network is highly robust, successfully distilling the necessary mutational variance regardless of whether a global mean or a localized residue-specific extraction strategy is applied.

In addition to pooling strategies, we evaluated the effect of the pretrained sequence embedding model on downstream performance, as detailed in Table S4. For this comparative analysis, we replaced the ESM-2 feature extractor with ESM-C [14]. This experiment was designed to

Table S3: Ablation of sequence embedding pooling strategies on the S2648 blind test. All models utilize ESM-2 alongside Phy and PSL features. Both Residue-specific Embedding and Mean Embedding strategies incorporate the WT sequence, MT sequence, and their element-wise difference.

| Sequence Pooling Strategy | PCC | RMSE |
|---|---|---|
| Mean Embedding of WT | 0.79 | 0.97 |
| Mean Embedding of MT | 0.79 | 0.97 |
| Residue-specific Embedding | 0.82 | 0.90 |
| Mean Embedding | 0.82 | 0.90 |

assess the sensitivity of SheafLapNet to the choice of sequence embedding model, rather than to provide an exhaustive benchmark of protein foundation models. When relying exclusively on sequence features, replacing ESM-2 with ESM-C improved the PCC from 0.76 to 0.80 and reduced the RMSE from 1.02 to 0.91. However, when sequence embeddings were integrated with physicochemical descriptors and PSL features, the full model showed a marginal reduction in RMSE from 0.90 to 0.89, while maintaining the same PCC of 0.82. These results suggest that the proposed topological and physicochemical descriptors provide complementary information beyond that captured by pretrained sequence embeddings.

Table S4: Ablation of pretrained sequence embedding models on the S2648 stability benchmark. The sequence pooling strategy is fixed to the mean embedding of WT and MT sequences with their element-wise difference.

| Features | Transformer | PCC | RMSE |
|---|---|---|---|
| Seq | ESM-2 | 0.76 | 1.02 |
| Seq | ESM-C | 0.80 | 0.91 |
| Seq + Phy + PSL | ESM-2 | 0.82 | 0.90 |
| Seq + Phy + PSL | ESM-C | 0.82 | 0.89 |

## S5 Supplementary Tables

To contextualize the performance of SheafLapNet against existing state-of-the-art models, we present the extended benchmark evaluations across both solubility and stability prediction tasks. Table S7 details the performance comparison on the independent blind test classification for protein solubility utilizing the PON-Sol2 dataset. For clarity, we isolate the two most comprehensive evaluation metrics, CPR and $GC^2$. Each metric is reported as its unnormalized value followed by its normalized value. Table S6 compiles the quantitative performance of SheafLapNet against established predictive models for mutation-induced stability changes $\Delta\Delta G$. The evaluation spans the S2648 dataset, utilized for 5-fold cross-validation, and the S350 independent blind test set. Performance is measured via the PCC and RMSE.

To address potential concerns regarding the computational overhead of the proposed framework, we quantified the execution time required for the neural network training and prediction phases across our benchmark datasets. It is important to note that these benchmarks specifically measure the performance of the deep learning module and exclude the preliminary feature extraction steps. As detailed in Table S8, the framework demonstrates high efficiency relative to the volume of data processed. All computational benchmarks were executed on a dedicated Linux server equipped with NVIDIA Tesla V100S GPUs (32 GB VRAM).

Table S5: Comparative benchmark evaluating the performance of various methods on the S669 independent blind test set. Results for baseline methods were collected from the corresponding publications or reported benchmark studies, while SheafLapNet results were obtained in this work using S2648 as the training set. Blank entries indicate that the corresponding metrics were not reported in the original source.

| Method | Total | | Forward | | Reverse | | Symmetry | |
|---|---|---|---|---|---|---|---|---|
| | PCC | RMSE | PCC | RMSE | PCC | RMSE | $r_{f-r}$ | $\langle\delta\rangle$ |
| **SheafLapNet** | **0.66** | **1.43** | **0.50** | **1.42** | **0.48** | **1.44** | **-0.99** | **-0.01** |
| PROSTATA [32] | 0.65 | 1.45 | 0.49 | 1.45 | 0.49 | 1.45 | -0.99 | -0.01 |
| ACDC-NN [3] | 0.61 | 1.5 | 0.46 | 1.49 | 0.45 | 1.5 | -0.98 | 0.02 |
| INPS-Seq [12] | 0.61 | 1.52 | 0.43 | 1.52 | 0.43 | 1.53 | -1.00 | 0.00 |
| PremPS [7] | 0.62 | 1.49 | 0.41 | 1.5 | 0.42 | 1.49 | -0.85 | 0.09 |
| ACDC-NN-Seq [24] | 0.59 | 1.53 | 0.42 | 1.53 | 0.42 | 1.53 | -1.00 | 0.00 |
| DDGun3D [23] | 0.57 | 1.61 | 0.43 | 1.6 | 0.41 | 1.62 | -0.97 | 0.05 |
| INPS3D [30] | 0.55 | 1.64 | 0.43 | 1.5 | 0.33 | 1.77 | -0.5 | 0.38 |
| THPLM [13] | 0.53 | 1.63 | 0.39 | 1.60 | 0.35 | 1.66 | -0.96 | -0.01 |
| ThermoNet [18] | 0.51 | 1.64 | 0.39 | 1.62 | 0.38 | 1.66 | -0.85 | 0.05 |
| DDGun [23] | 0.57 | 1.74 | 0.41 | 1.72 | 0.38 | 1.75 | -0.96 | 0.05 |
| MAESTRO [16, 17] | 0.44 | 1.8 | 0.5 | 1.44 | 0.2 | 2.1 | -0.22 | 0.57 |
| ThermoMPNN [10] | 0.43 | 1.52 | | | | | | |
| Dynamut [28] | 0.5 | 1.65 | 0.41 | 1.6 | 0.34 | 1.69 | -0.58 | 0.06 |
| PoPMuSiC [9] | 0.46 | 1.82 | 0.41 | 1.51 | 0.24 | 2.09 | -0.32 | 0.69 |
| DUET [25] | 0.41 | 1.86 | 0.41 | 1.52 | 0.23 | 2.14 | -0.12 | 0.67 |
| I-Mutant3.0-Seq [5] | 0.37 | 1.91 | 0.34 | 1.54 | 0.22 | 2.22 | -0.48 | 0.76 |
| SDM [34] | 0.32 | 1.93 | 0.41 | 1.67 | 0.13 | 2.16 | -0.4 | 0.4 |
| mCSM [26] | 0.37 | 1.96 | 0.36 | 1.54 | 0.22 | 2.3 | -0.05 | 0.85 |
| Dynamut2 [29] | 0.36 | 1.9 | 0.34 | 1.58 | 0.17 | 2.16 | 0.03 | 0.64 |
| I-Mutant3.0 [5] | 0.32 | 1.96 | 0.36 | 1.52 | 0.15 | 2.32 | -0.06 | 0.81 |
| Rosetta [15] | 0.47 | 2.69 | 0.39 | 2.7 | 0.4 | 2.68 | -0.72 | 0.61 |
| FoldX [31] | 0.31 | 2.39 | 0.22 | 2.3 | 0.22 | 2.48 | -0.2 | 0.34 |
| SAAFEC-SEQ [19] | 0.26 | 2.02 | 0.36 | 1.54 | -0.01 | 2.4 | -0.03 | 0.83 |
| MUpro [8] | 0.32 | 2.03 | 0.25 | 1.61 | 0.20 | 2.38 | -0.32 | 0.95 |

## S6 Supplementary Figures

Table S6: Comparison of SheafLapNet's predictive performance with the reported PCC and RMSE for the mutation-induced protein stability change prediction datasets. Existing results are referenced from [4] unless otherwise stated. [a] Results obtained from [34]. [b] Results obtained from [27]. [c] According to Ref. [27] the data from the online server has PCC (RMSE) of 0.59 (1.28) and 0.70 (1.13) for INPS and mCSM respectively in the task of S350 set. The $n$ column denotes the number of mutation samples successfully processed by each method.

| S2648 | | | | S350 | | | |
|---|---|---|---|---|---|---|---|
| Method | $n$ | PCC | RMSE | Method | $n$ | PCC | RMSE |
| **SheafLapNet** | **2648** | **0.82** | **0.84** | **SheafLapNet** | **350** | **0.82** | **0.90** |
| TNet-MP-2 | 2648 | 0.77 | 0.94 | TNet-MP-2 | 350 | 0.81 | 0.94 |
| STRUM[b] | 2647 | 0.77 | 0.94 | STRUM[b] | 350 | 0.79 | 0.98 |
| TNet-MP-1 | 2648 | 0.72 | 1.02 | TNet-MP-1 | 350 | 0.74 | 1.07 |
| mCSM[b,c] | 2643 | 0.69 | 1.07 | mCSM[b,c] | 350 | 0.73 | 1.08 |
| PoPMuSiC2.0[b] | 2647 | 0.61 | 1.17 | INPS[b,c] | 350 | 0.68 | 1.25 |
| I-Mutant3.0[b] | 2636 | 0.60 | 1.19 | PoPMuSiC2.0[b] | 350 | 0.67 | 1.16 |
| INPS [b,c] | 2648 | 0.56 | 1.26 | PoPMuSiC 1.0[a] | 350 | 0.62 | 1.23 |
| | | | | I-Mutant3.0[b] | 338 | 0.53 | 1.35 |
| | | | | Dmutant[a] | 350 | 0.48 | 1.38 |
| | | | | Automute[a] | 315 | 0.46 | 1.42 |
| | | | | CUPSAT[a] | 346 | 0.37 | 1.46 |
| | | | | Eris[a] | 334 | 0.35 | 1.49 |
| | | | | I-Mutant 2.0[a] | 346 | 0.29 | 1.50 |

To transparently evaluate predictive capabilities across specific residue transitions, we generated an explicit error matrix alongside the sample distribution. Figure S3 contrasts the raw mutation sample counts with the corresponding MSE for every wild-type and mutant amino acid pairing. This paired analysis reveals consistent architectural reliability across most substitution types, particularly within well-represented transitions involving Alanine, Valine, and Isoleucine. Conversely, elevated error rates are sparsely distributed and tightly linked to extreme biochemical transitions lacking sufficient training representation, such as specific shifts between Lysine and Valine or Methionine and Threonine. These isolated high-error pockets directly correspond to minimal sample counts, confirming that predictive vulnerabilities are not systemic but constrained to specific data-sparse evolutionary shifts.

Expanding upon this localized residue evaluation, we grouped the mutations to assess broader biochemical patterns. Figure S4(a) presents a heatmap illustrating the PCC across physicochemical mutation pairs. For this granular assessment, all wild-type and mutated residues were categorized into six non-overlapping functional groups, namely Hydrophobic, Polar, Negatively Charged, Positively Charged, Alanine, and Non-Alanine. Figure S4(b) features a grid of detailed scatter plots mapping the predicted against the experimental stability changes for six representative transition categories, specifically Negatively Charged to Positively Charged, Polar to Hydrophobic, Non-Alanine to Alanine, Positively Charged to Negatively Charged, Hydrophobic to Polar, and Alanine to Non-Alanine. Each individual plot incorporates the calculated PCC and RMSE to provide a strict quantitative measure of the predictive capability for that specific mutation type.

Table S7: Performance of SheafLapNet compared with existing models on the PON-Sol2 independent blind test classification for solubility prediction. Except for SheafLapNet, performance metrics for all baseline methods were retrieved from [33].

| | CPR | | $GC^2$ | |
|---|---|---|---|---|
| Model | Unnorm. | Norm. | Unnorm. | Norm. |
| **SheafLapNet** | **0.737** | **0.638** | **0.270** | **0.254** |
| TopGBT | 0.707 | 0.562 | 0.205 | 0.184 |
| PON-Sol2 | 0.671 | 0.545 | 0.181 | 0.157 |
| SODA (17 as Threshold) | 0.382 | 0.356 | 0.016 | 0.016 |
| SODA (5 as Threshold) | 0.381 | 0.341 | 0.041 | 0.045 |
| SODA (10 as Threshold) | 0.375 | 0.347 | 0.022 | 0.022 |
| PON-Sol | 0.356 | 0.389 | 0.010 | 0.011 |
| SODA | 0.282 | 0.247 | NaN | NaN |

Table S8: Summary of specific training and evaluation datasets, alongside the required computational execution time, for each stability and solubility prediction task. Time is reported in minutes and encompasses the complete training and prediction cycle across all ten independently seeded models.

| Protein Stability Prediction | | | |
|---|---|---|---|
| Task / Protocol | Training Dataset | Evaluation Dataset | Time (min) |
| 5-fold Cross-Validation | S2648 | S2648 | 18.10 |
| Independent Blind Test | S2648 (w/o S350) | S350 | 1.09 |
| Independent Blind Test | S2648 | S669 | 1.40 |
| **Protein Solubility Prediction** | | | |
| Task / Protocol | Training Dataset | Evaluation Dataset | Time (min) |
| 10-fold Cross-Validation | PON-Sol2 | PON-Sol2 | 10.34 |
| Independent Blind Test | PON-Sol2 (Train Split) | PON-Sol2 (Test Split) | 1.50 |

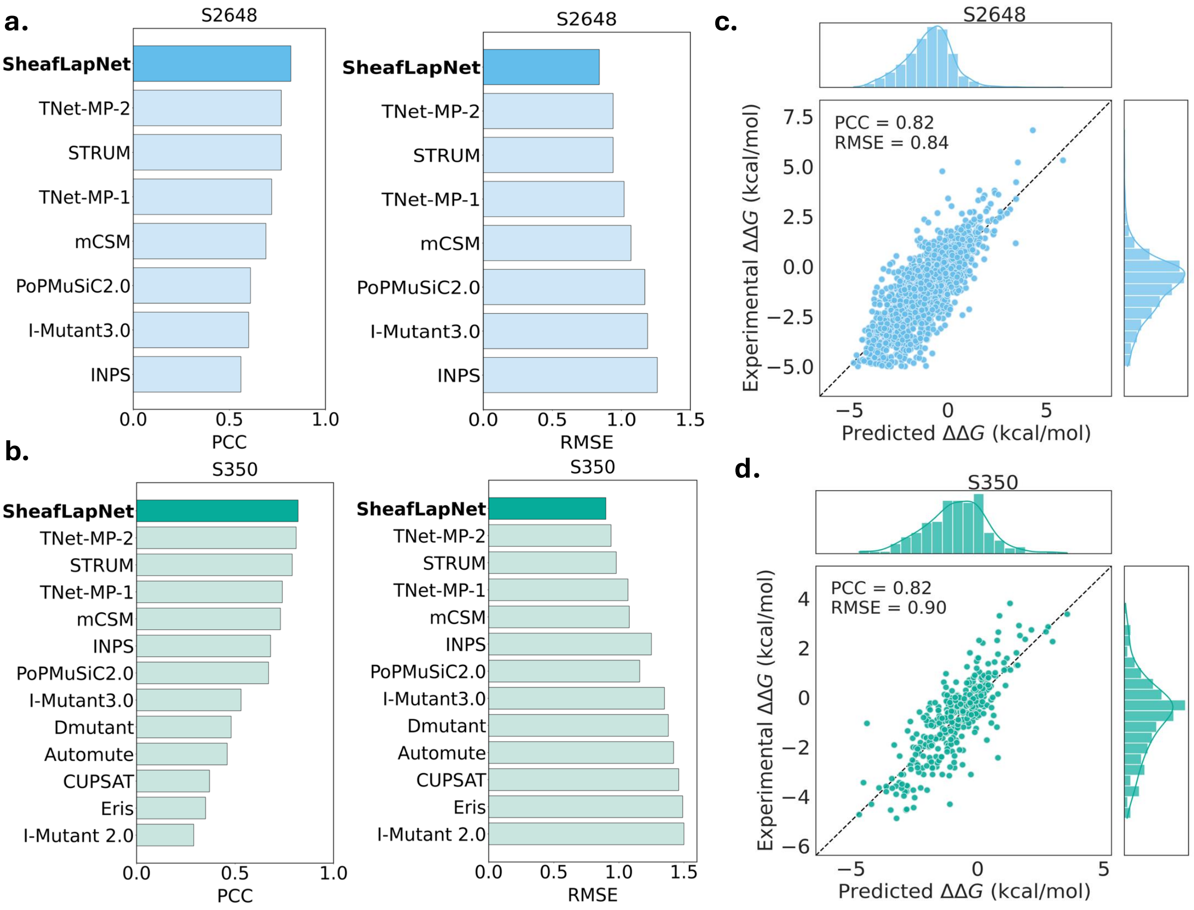


Figure S2: Illustration of model performance on the S350 and S2648 datasets in protein stability changes upon mutation. (a). 5-fold cross-validation performance of SheafLapNet for S2648 dataset compared to existing state-of-the-art models [4, 27, 34]. (b). Blind test performance of SheafLapNet for S350 dataset compared to existing state-of-the-art models [4, 5, 27, 34]. (c). Comparison of experimental protein stability changes with predicted ones from SheafLapNet for the S2648 dataset. (d). Comparison of experimental protein stability changes with predicted ones from SheafLapNet for S350 dataset. Note: An average of 99.58% of the validation mutations in the 5-fold cross-validation (a, c) and 346 out of 350 mutations in the blind test (b, d) share $> 25\%$ sequence identity with their respective training sets.

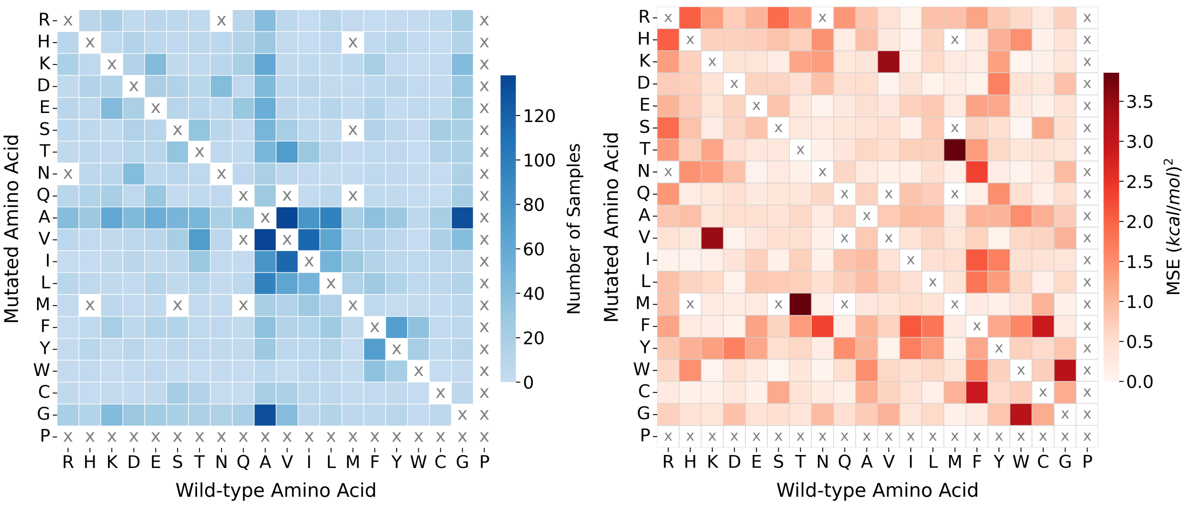


Figure S3: Distribution of mutation samples and corresponding MSE across amino acid substitutions in the S2648 dataset. The left panel displays the sample count for each specific substitution pair, where the x-axis corresponds to the wild-type residue and the y-axis denotes the mutant residue. The right panel illustrates the MSE for these identical substitution pairs to highlight the predictive strengths and vulnerabilities of the model. In both panels, an X indicates the deliberate exclusion or absence of mutation samples for that specific transition.

# S7 Auxiliary Descriptors

To complement the topological representations derived from the Persistent Sheaf Laplacian, we incorporated a comprehensive set of auxiliary features designed to capture essential physicochemical interactions. These descriptors are categorized into atom-level and residue-level features.

## S7.1 Atom-Level Features

To achieve a high-resolution representation of the molecular environment, we employ a hierarchical categorization strategy based on element type, spatial proximity, and mutation state. Atoms are first classified into seven distinct element groups: Carbon (C), Nitrogen (N), Oxygen (O), Sulfur (S), Hydrogen (H), heavy atoms, and all atoms. To capture interactions at varying scales, these element groups are further subdivided by spatial region, specifically targeting atoms at the mutation site, atoms within a 10 Å neighborhood radius, and all atoms. Finally, to explicitly encode mutation-induced alterations, feature vectors are generated for the wild-type structure, the mutant structure, and the difference between them.

Based on these categorizations, we compute four primary classes of physicochemical descriptors:

1. **Solvent-excluded surface area.** Atom-level solvent-excluded surface areas are calculated using the ESES software [21]. For each defined atom group, the surface areas of individual atoms are aggregated to generate a single feature. This yields a total of $7 \times 3 \times 3 = 63$ features.

2. **Partial charge distribution.** Partial charges are computed using the PDB2PQR software [11] with the AMBER force field. To quantify the electrostatic distribution, we calculate two metrics for each atom group: the sum of partial charges ($\sum q_i$) and the sum of absolute partial charges ($\sum |q_i|$). This results in $7 \times 3 \times 2 \times 3 = 126$ features.

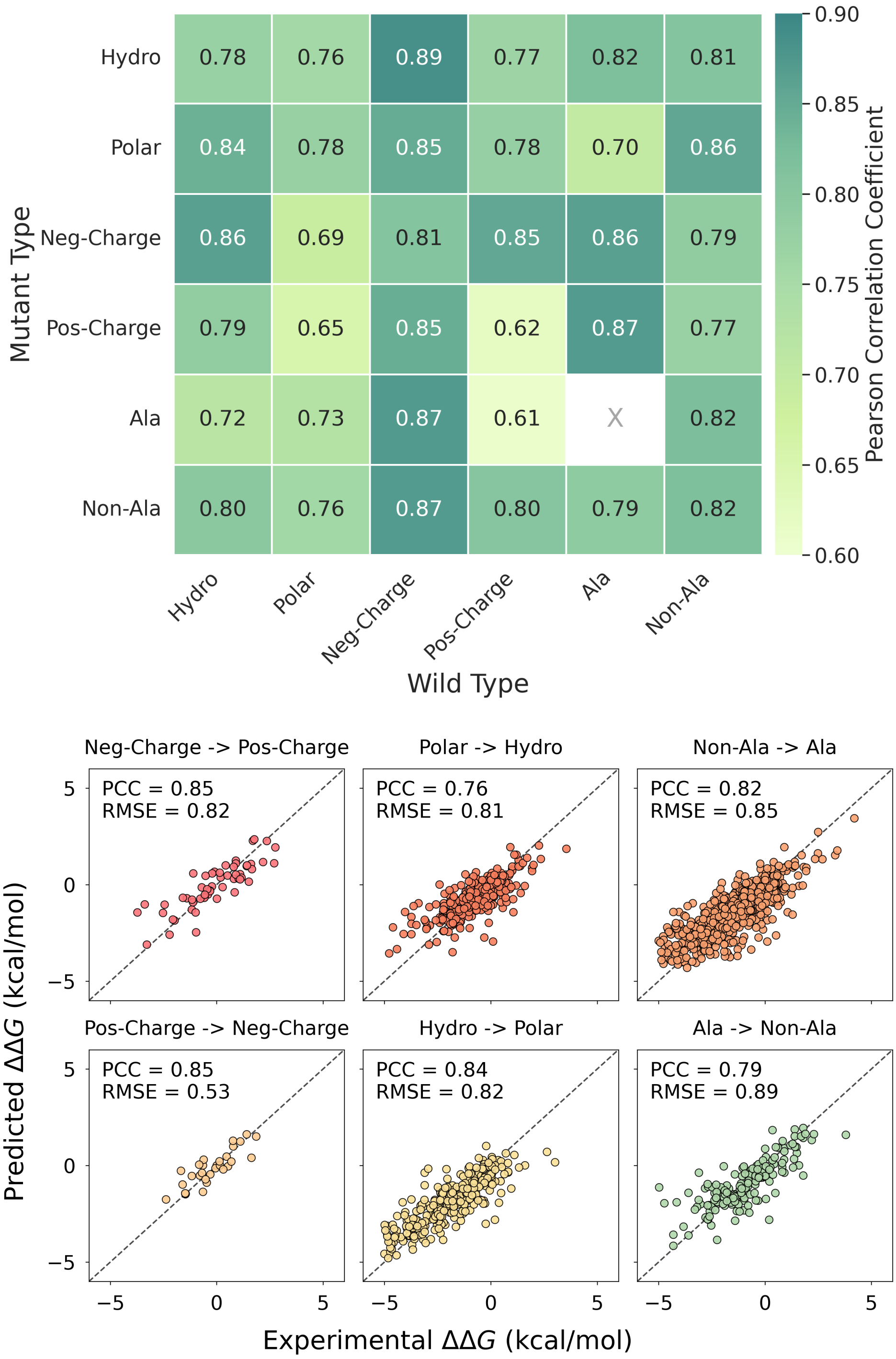


Figure S4: The correlation analysis of predicted versus experimental protein stability changes. The top panel presents a heatmap detailing the PCC across all possible physicochemical transitions between wild-type and mutated residues. The white cell marked with an X indicates the deliberate exclusion of synonymous Alanine to Alanine mutations. The bottom panel provides scatter plots for six representative mutation pairs to illustrate predictive accuracy and error distribution across diverse biochemical environments.

3. **Coulomb interactions.** The Coulomb energy of the $i$-th atom is derived from the sum

of pairwise electrostatic interactions with every other atom $j$ in the system:

$$C_i = \sum_{j \neq i} \frac{q_i q_j}{r_{ij}}, \tag{S7}$$

where $q_i$ and $q_j$ are partial charges, and $r_{ij}$ is the Euclidean interatomic distance. We consider five specific element groups (C, N, O, S, and Heavy atoms) and compute both the signed Coulomb energy and its absolute value. This yields $5 \times 3 \times 2 \times 3 = 90$ features.

4. **Van der Waals interactions.** The van der Waals energy of the $i$-th atom is computed using the Lennard-Jones potential with a 40 Å cutoff:

$$V_i = \sum_{j \neq i} \epsilon \left[ \left( \frac{r_i + r_j}{r_{ij}} \right)^{12} - 2 \left( \frac{r_i + r_j}{r_{ij}} \right)^{6} \right], \tag{S8}$$

where $r_i$ and $r_j$ denote van der Waals radii and $\epsilon = 1$ represents the potential well depth. Similar to Coulomb interactions, calculations are performed for five element groups, resulting in $5 \times 3 \times 3 = 45$ features.

5. **Electrostatic solvation energy.** The electrostatic solvation free energy for each atom is calculated using the Poisson-Boltzmann model via the MIBPB software [6]. The solvation free energies of all atoms within the same specific groups are summed to generate a single feature per group. This process yields a total of $7 \times 3 \times 3 = 63$ features.

### S7.2 Residue-Level Features

1. **Mutation neighborhood micro-environment.** Residues within a 10 Å radius of the mutation site are characterized to capture the local physicochemical environment. These neighboring residues are classified into five categories: hydrophobic, polar, positively charged, negatively charged, and special cases. Both the count and percentage for each category are calculated, yielding 10 features. Additionally, the sum, mean, and variance of residue volume, surface area, molecular weight, and hydropathy score are computed. This aggregation results in 12 features.

2. **pKa shifts.** The pKa values of seven ionizable amino acids (ASP, GLU, ARG, LYS, HIS, CYS, TYR) are computed using the PROPKA software [2]. We extract global statistics including the maximum, minimum, and net pKa shifts between wild-type and mutant structures. Specific pKa values for the mutation site, N-terminus, and C-terminus are recorded for the wild-type, mutant, and their difference. Furthermore, the net and absolute pKa shifts within each of the seven residue groups are included.

3. **Position specific scoring matrix (PSSM).** Evolutionary conservation features are derived from the PSSM of the mutation site, generated by PSI-BLAST [1]. We extract conservation scores for the wild-type residue, the mutant residue, their difference, and the row total from both log-odds and weighted probability matrices. Additionally, information content metrics are included.

4. **Secondary structure.** The SPIDER software [36] is used to predict backbone torsion angles $(\phi, \psi)$ and the probability of the residue adopting a coil, $\alpha$-helix, or $\beta$-strand conformation. Combined with DSSP structural assignments, these features are computed for the wild-type, mutant, and their difference.

## S8 Software and Resources

- The 3D protein structures for S2648 and S350 datasets were obtained from the Protein Data Bank, while 3D protein structures for PON-Sol2 dataset were obtained from the structures generated in [33]. Mutant proteins are then generated from the Jackal software [35].
- The PDB2PQR software [11] with the AMBER force field is used to generate the partial charges of atoms.
- The ESES software [21] is used to compute the solvent excluded surface area of atoms.
- The MIBPB package [6] is used to compute the electrostatic solvation free energy of atoms.
- The pKa values are calculated via the PROPKA package [2].
- The PSI-BLAST software [1] with the UniRef50 database is used to compute the PSSM.
- The SPIDER2 software [36] is used to compute secondary structure features.
- For persistent Sheaf Laplacian descriptors, the GUDHI software library [22] is used to generate both VR complexes and Alpha complexes in the filtration process. The VR complexes and Alpha complexes are then used to construct the persistent Laplacian matrices.
- The ESM-2 model [20] is used to generate the evolutionary sequence features.

## References


[1] S. F. Altschul, T. L. Madden, A. A. Schäffer, J. Zhang, Z. Zhang, W. Miller, and D. J. Lipman. Gapped BLAST and PSI-BLAST: a new generation of protein database search programs. *Nucleic Acids Research*, 25(17):3389–3402, 1997.

[2] D. C. Bas, D. M. Rogers, and J. H. Jensen. Very fast prediction and rationalization of pKa values for protein–ligand complexes. *Proteins: Structure, Function, and Bioinformatics*, 73(3):765–783, 2008.

[3] S. Benevenuta, C. Pancotti, P. Fariselli, G. Birolo, and T. Sanavia. An antisymmetric neural network to predict free energy changes in protein variants. *Journal of Physics D: Applied Physics*, 54(24):245403, 2021.

[4] Z. Cang and G.-W. Wei. TopologyNet: Topology based deep convolutional and multi-task neural networks for biomolecular property predictions. *PLoS computational biology*, 13(7):e1005690, 2017.

[5] E. Capriotti, P. Fariselli, and R. Casadio. I-Mutant2. 0: predicting stability changes upon mutation from the protein sequence or structure. *Nucleic acids research*, 33(suppl_2):W306–W310, 2005.

[6] D. Chen, Z. Chen, C. Chen, W. Geng, and G.-W. Wei. MIBPB: a software package for electrostatic analysis. *Journal of computational chemistry*, 32(4):756–770, 2011.

[7] Y. Chen, H. Lu, N. Zhang, Z. Zhu, S. Wang, and M. Li. PremPS: Predicting the impact of missense mutations on protein stability. *PLoS computational biology*, 16(12):e1008543, 2020.

[8] J. Cheng, A. Randall, and P. Baldi. Prediction of protein stability changes for single-site mutations using support vector machines. *Proteins: Structure, Function, and Bioinformatics*, 62(4):1125–1132, 2006.

[9] Y. Dehouck, J. M. Kwasigroch, D. Gilis, and M. Rooman. PoPMuSiC 2.1: a web server for the estimation of protein stability changes upon mutation and sequence optimality. *BMC bioinformatics*, 12(1):151, 2011.

[10] H. Dieckhaus, M. Brocidiacono, N. Z. Randolph, and B. Kuhlman. Transfer learning to leverage larger datasets for improved prediction of protein stability changes. *Proceedings of the national academy of sciences*, 121(6):e2314853121, 2024.

[11] T. J. Dolinsky, J. E. Nielsen, J. A. McCammon, and N. A. Baker. PDB2PQR: an automated pipeline for the setup of poisson–boltzmann electrostatics calculations. *Nucleic acids research*, 32(suppl_2):W665–W667, 2004.

[12] P. Fariselli, P. L. Martelli, C. Savojardo, and R. Casadio. INPS: predicting the impact of non-synonymous variations on protein stability from sequence. *Bioinformatics*, 31(17):2816–2821, 2015.

[13] J. Gong, J. Wang, X. Zong, Z. Ma, and D. Xu. Prediction of protein stability changes upon single-point variant using 3D structure profile. *Computational and structural biotechnology journal*, 21:354–364, 2023.

[14] T. Hayes, R. Rao, H. Akin, N. J. Sofroniew, D. Oktay, Z. Lin, R. Verkuil, V. Q. Tran, J. Deaton, M. Wiggert, et al. Simulating 500 million years of evolution with a language model. *Science*, 387(6736):850–858, 2025.

[15] E. H. Kellogg, A. Leaver-Fay, and D. Baker. Role of conformational sampling in computing mutation-induced changes in protein structure and stability. *Proteins: Structure, Function, and Bioinformatics*, 79(3):830–838, 2011.

[16] J. Laimer, J. Hiebl-Flach, D. Lengauer, and P. Lackner. MAESTROweb: a web server for structure-based protein stability prediction. *Bioinformatics*, 32(9):1414–1416, 2016.

[17] J. Laimer, H. Hofer, M. Fritz, S. Wegenkittl, and P. Lackner. MAESTRO-multi agent stability prediction upon point mutations. *BMC bioinformatics*, 16(1):116, 2015.

[18] B. Li, Y. T. Yang, J. A. Capra, and M. B. Gerstein. Predicting changes in protein thermodynamic stability upon point mutation with deep 3d convolutional neural networks. *PLoS computational biology*, 16(11):e1008291, 2020.

[19] G. Li, S. K. Panday, and E. Alexov. SAAFEC-SEQ: a sequence-based method for predicting the effect of single point mutations on protein thermodynamic stability. *International journal of molecular sciences*, 22(2):606, 2021.

[20] Z. Lin, H. Akin, R. Rao, B. Hie, Z. Zhu, W. Lu, N. Smetanin, R. Verkuil, O. Kabeli, Y. Shmueli, et al. Evolutionary-scale prediction of atomic-level protein structure with a language model. *Science*, 379(6637):1123–1130, 2023.

[21] B. Liu, B. Wang, R. Zhao, Y. Tong, and G.-W. Wei. ESES: Software for e ulerian solvent excluded surface, 2017.

[22] C. Maria, J.-D. Boissonnat, M. Glisse, and M. Yvinec. The gudhi library: Simplicial complexes and persistent homology. In *International congress on mathematical software*, pages 167–174. Springer, 2014.

[23] L. Montanucci, E. Capriotti, Y. Frank, N. Ben-Tal, and P. Fariselli. DDGun: an untrained method for the prediction of protein stability changes upon single and multiple point variations. *BMC bioinformatics*, 20(Suppl 14):335, 2019.

[24] C. Pancotti, S. Benevenuta, G. Birolo, V. Alberini, V. Repetto, T. Sanavia, E. Capriotti, and P. Fariselli. Predicting protein stability changes upon single-point mutation: a thorough comparison of the available tools on a new dataset. *Briefings in Bioinformatics*, 23(2):bbab555, 2022.

[25] D. E. Pires, D. B. Ascher, and T. L. Blundell. DUET: a server for predicting effects of mutations on protein stability using an integrated computational approach. *Nucleic acids research*, 42(W1):W314–W319, 2014.

[26] D. E. Pires, D. B. Ascher, and T. L. Blundell. mCSM: predicting the effects of mutations in proteins using graph-based signatures. *Bioinformatics*, 30(3):335–342, 2014.

[27] L. Quan, Q. Lv, and Y. Zhang. STRUM: structure-based prediction of protein stability changes upon single-point mutation. *Bioinformatics*, 32(19):2936–2946, 2016.

[28] C. H. Rodrigues, D. E. Pires, and D. B. Ascher. DynaMut: predicting the impact of mutations on protein conformation, flexibility and stability. *Nucleic acids research*, 46(W1):W350–W355, 2018.

[29] C. H. Rodrigues, D. E. Pires, and D. B. Ascher. DynaMut2: assessing changes in stability and flexibility upon single and multiple point missense mutations. *Protein Science*, 30(1):60–69, 2021.

[30] C. Savojardo, P. Fariselli, P. L. Martelli, and R. Casadio. INPS-MD: a web server to predict stability of protein variants from sequence and structure. *Bioinformatics*, 32(16):2542–2544, 2016.

[31] J. Schymkowitz, J. Borg, F. Stricher, R. Nys, F. Rousseau, and L. Serrano. The FoldX web server: an online force field. *Nucleic acids research*, 33(suppl_2):W382–W388, 2005.

[32] D. Umerenkov, F. Nikolaev, T. I. Shashkova, P. V. Strashnov, M. Sindeeva, A. Shevtsov, N. V. Ivanisenko, and O. L. Kardymon. PROSTATA: a framework for protein stability assessment using transformers. *Bioinformatics*, 39(11):btad671, 2023.

[33] J. Wee, J. Chen, K. Xia, and G.-W. Wei. Integration of persistent laplacian and pre-trained transformer for protein solubility changes upon mutation. *Computers in biology and medicine*, 169:107918, 2024.

[34] C. L. Worth, R. Preissner, and T. L. Blundell. SDM—a server for predicting effects of mutations on protein stability and malfunction. *Nucleic acids research*, 39(suppl_2):W215–W222, 2011.

[35] J. Z. Xiang and B. Honig. Jackal: A protein structure modeling package. *Columbia University and Howard Hughes Medical Institute, New York*, 2002.

[36] Y. Yang, R. Heffernan, K. Paliwal, J. Lyons, A. Dehzangi, A. Sharma, J. Wang, A. Sattar, and Y. Zhou. SPIDER2: a package to predict secondary structure, accessible surface area, and main-chain torsional angles by deep neural networks. In *Prediction of protein secondary structure*, pages 55–63. Springer, 2016.
